# Conformational Toggling of Yeast Iso-1-Cytochrome *c* in the Oxidized and Reduced States

**DOI:** 10.1371/journal.pone.0027219

**Published:** 2011-11-08

**Authors:** Wenxian Lan, Zhonghua Wang, Zhongzheng Yang, Jing Zhu, Tianlei Ying, Xianwang Jiang, Xu Zhang, Houming Wu, Maili Liu, Xiangshi Tan, Chunyang Cao, Zhong-Xian Huang

**Affiliations:** 1 State Key Laboratory of Bioorganic and Natural Products Chemistry, Shanghai Institute of Organic Chemistry, Chinese Academy of Sciences, Shanghai, China; 2 Chemical Biology Laboratory, Department of Chemistry, Fudan University, Shanghai, China; 3 State Key Laboratory of Magnetic Resonance and Atomic and Molecular Physics, Wuhan Institute of Physics and Mathematics, Chinese Academy of Sciences, Wuhan, China; Russian Academy of Sciences, Institute for Biological Instrumentation, Russian Federation

## Abstract

To convert cyt *c* into a peroxidase-like metalloenzyme, the P71H mutant was designed to introduce a distal histidine. Unexpectedly, its peroxidase activity was found even lower than that of the native, and that the axial ligation of heme iron was changed to His71/His18 in the oxidized state, while to Met80/His18 in the reduced state, characterized by UV-visible, circular dichroism, and resonance Raman spectroscopy. To further probe the functional importance of Pro71 in oxidation state dependent conformational changes occurred in cyt *c*, the solution structures of P71H mutant in both oxidation states were determined. The structures indicate that the half molecule of cyt *c* (aa 50–102) presents a kind of “zigzag riveting ruler” structure, residues at certain positions of this region such as Pro71, Lys73 can move a big distance by altering the tertiary structure while maintaining the secondary structures. This finding provides a molecular insight into conformational toggling in different oxidation states of cyt *c* that is principle significance to its biological functions in electron transfer and apoptosis. Structural analysis also reveals that Pro71 functions as a key hydrophobic patch in the folding of the polypeptide of the region (aa 50–102), to prevent heme pocket from the solvent.

## Introduction

Cytochrome *c* (cyt *c*) is a class of electron transport hemoproteins by the covalent attachment of a heme prosthetic group to a peptide through two thioether bonds between the vinyl groups of heme ring and cysteine sulfhydryls of the CXXCH heme-binding motif of the protein. The axial coordination of heme iron plays key roles in determining redox, electron transfer, and other properties of cyt *c* [1]–[3]. Methionine and histidine are the invariable axial ligands of native mitochondrial cyt *c*, other variants have these ligands substituted or deleted [1], [4]. Several factors that were closely associated with the changes in axial coordination had been reported as pH linked conformational changes (the native form versus the alkaline form) [5]–[7], redox “switched” changes (folded versus unfolded form) [8]–[13], and the refolding of cyt *c* [14]–[18]. These coordination changes and rearrangements were suggested as a mechanism for converting redox energy into conformational energy, or as a switch from an intermediate His/His coordinated form to the native Met/His form [15]–[19].

**Figure 1:**
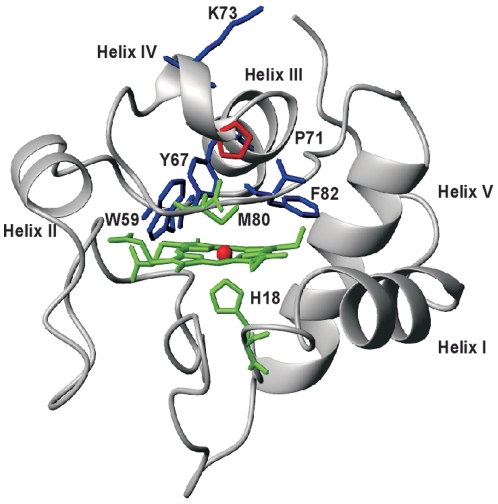
Structure of the heme region of cyt *c* and the location of Pro71 in the structure (pdb code: 1YIC). The selected amino acids including His18, Tyr67, Met80, Phe82 and Pro71 were shown.

In yeast iso-1 cyt *c*, the conserved residue Pro71 locates at the C-terminus of helix III (aa 60–70) and in the beginning of helix IV (71–74) (Figure 1). Without amide proton, Pro71 is unable to form a hydrogen bond with the carbonyl group of Tyr67, so that the helix III cannot be elongated. In the X-ray structure of yeast cyt *c* (pdb code 1YCC), the carbonyl group of Pro71 forms a hydrogen bond with Ile75 N atom in Helix IV. Therefore, Pro71 plays important roles in directing the proper folding of the polypeptide chain in this region [20]–[21], which occludes the main chain and side chain atoms of Pro71 from bulk solvent. By packing against the side chains of Tyr67, Met80 and Phe82, Pro71 functions as a hydrophobic patch in the whole folding of cyt *c*. Due to the flexibility of polypeptide of region from Pro71 to Gly83 in the oxidized cyt *c*, it was suggested to play functional roles in oxidation state dependent conformational changes [22].

In this paper, to study the function of Pro71, the original pBTR2 plasmid encoding genes of the yeast iso-1 cyt *c* (*CYC*1) and yeast cyt *c* heme lyase (*CYC*3) contains K72A/C102T mutations. The change from Cys102 to Thr102 was used to prevent disulfide dimerization of the protein and protein auto-reduction [7], [23]–[24], which may alter the cytochrome's thermodynamic and spectroscopic parameters. The mutation from Lys72 to Ala72 was utilized to prevent this residue from serving as a ligand in the alkaline form of cyt *c* at pH 7.0. For the sake of simplicity, throughout this paper, the double-site K72A/C102T variant was used as the reference and referred to as native yeast iso-1 cyt *c*. The triple mutant P71H/K72A/C102T used in this study was referred to as the P71H variant.

The cyt *c* P71H mutant was originally designed to convert an electron transfer metalloprotein, cyt *c*, to a catalytic metalloenzyme – peroxidase. The main differences on the structural element between cyt *c* and peroxidase are those listed as follows: (1) the 6^th^ axial ligand of heme in peroxidase is vacant, which is for loading of substrate; (2) in the heme pocket of peroxidase there is a distal histidine facilitating the formation of compound I intermediate; (3) in the heme pocket the existence of a distal arginine to assist this process. As shown in Figure 1, the Pro71 is located at the distal position of heme pocket. If it is replaced by histidine, the distance between Nε atom of His71 and heme ferric iron is 5.62 Å which is very close to the distances of 5.84 Å and 5.55 Å between N_ε_ atom of the distal histidine and heme iron ion in Horseradish Peroxidase (HRP, pdb codes 1H5A) and Cytochrome *c* Peroxidase (C*c*P, pdb codes 2CYP) [25], [26], respectively. So, if residue Pro71 of cyt *c* is replaced by histidine by using site-directed mutagenesis, the cyt *c* P71H mutant shall be supposed to have peroxidase activity.

Out of our expectation, the cyt *c* P71H mutant was finally found to have no peroxidase activity at all (Method S1 and Figure S1), this was confirmed by using UV-visible spectra, circular diachroism (CD) spectra, and Raman resonance spectroscopy. It seems that the histidine introduced at position 71 likely acted as a strong sixth axial ligand of heme iron in the oxidized state at pH 7.0, while in the reduced state of the P71H variant at pH 7.0, the axial iron ligands switched to His18 and Met80, as did in the native cyt *c*. This kind of axial ligand conformation switching of cyt *c* was claimed in the F82H and K79H mutants of cyt *c* [10]–[13], [19], where His82 and His79 were thought to be the histidine on the distal side of the heme in the oxidized state of yeast iso-1 cyt *c*. However, there was no solid structural evidence to support this kind of conformational toggling. So, by using conventional two dimensional ^1^H-^1^H NMR methods [27], we determined the NMR solution structures of the cyt *c* P71H variant in both oxidized and reduced states.

## Results

### UV-visible and CD spectroscopy

**Figure 2:**
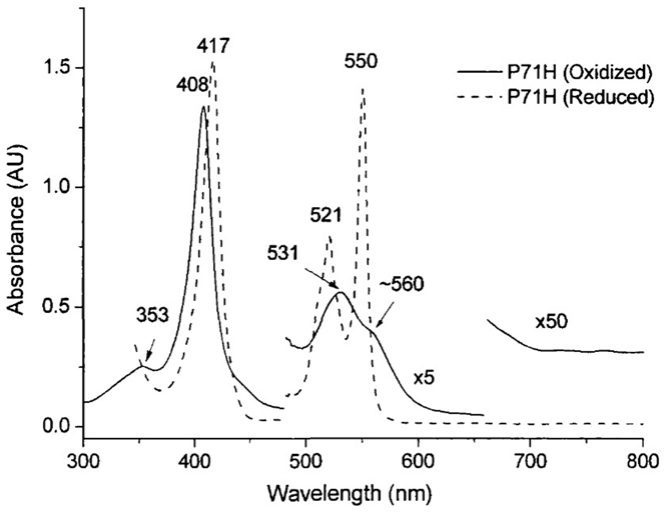
UV-Visible absorption spectra of cyt *c* P71H variant in the oxidized (solid line) and reduced (dashed line) states. Conditions: 100 mM phosphate buffer, pH 7.0.

As shown in Figures 2, Figures S2 and S3, and Table 1, the ferric form of P71H mutant demonstrates visible absorption maximal value at 408 nm (Soret band), 530 nm (α+β bands) and 353 nm (δ band), indicating that the oxidized P71H mutant has a 6-coordination low spin heme iron, almost identical to these of the native cyt *c* except the blue shift occurred at δ band. However, the absorption near 695 nm, characteristic sulfur (methionine)-Fe(III) charge transfer band of the native cyt *c* [28], is absent in the oxidized P71H variant, suggesting that the sixth ligand was no longer Met80 in the oxidized form of P71H mutant. These absorption bands of the oxidized P71H mutant were consistent with those of horse heart cyt *c* M80H mutant with His/His as its axial ligands [3], [4]. Interestingly, the UV-visible absorptions of the reduced P71H mutant are similar to these of reduced native cyt *c*, but different from these of the reduced horse heart cyt *c* M80H mutant [4], which infers that in the reduced P71H mutant the axial ligands of heme iron switch back to Met80/His18, similar to that in the reduced native cyt *c*.

**Table 1:** UV-visible absorption data for native cyt *c* and its P71H mutant.

protein	λ_max_, nm			
	Charge transfer	α/β	Scort (γ)	δ
oxidized native cyt *c*	∼695	530	410	360
oxidized P71H		530	408	353
reduced native cyt *c*		550/521	416	
reduced P71H		550/521	417	

The CD spectra in far UV region (Figures S4-A and S4-B) indicated that the helical content of the P71H mutant in oxidized state was about 35%, close to the calculated helical content 40% for yeast iso-1 cyt *c* (pdb code 1YCC), suggesting that the secondary structure in the P71H variant was mostly maintained. However, the CD spectrum of oxidized P71H variant was much different from that of the oxidized native cyt *c* (Figure S4-C), but consistent with that of oxidized yeast iso-1 cyt *c* F82H mutant [10]–[13], [19], especially in the negative absorption at 418 nm, which is characteristic of the interaction between Met80 and heme iron [29]–[31]. The almost identical CD spectra of reduced P71H mutant with the reduced native cyt *c* were seen in Figures S4-C and S4-D, consistent with the results from its UV spectrum above.

### Resonance Raman Spectroscopy

To further confirm the axial ligand coordination in both oxidized and reduced P71H mutant, the resonance Raman (RR) spectra were measured for native cyt *c* and its P71H mutant (Figures S5 and S6, and Table S1). The assigned RR data of ν_3_ 1501 cm^−1^ and ν_4_ 1370 cm^−1^ of native cyt *c* are identical to the reported data [19], [32]. The absorption values of ν_3_ and ν_4_ of the P71H mutant suggested that it also had 6-coordination low spin heme iron, consistent with the result from UV-visible spectroscopy. In the RR spectra of oxidized states, the main difference between the native cyt *c* and P71H mutant laid in the absorptions in ν(C_α_-S), δ(C_β_C_α_S) and δ(C_β_C_α_C_d_), which were assigned as the characteristic absorption of thioether bonds between heme and the peptide, and the absorption of the propionate group of heme, respectively. The absorptions in ν(C_α_-S), δ(C_β_C_α_S) and δ(C_β_C_α_C_d_) of the P71H mutant are almost similar to these of the cyt *c* F82H mutant [33]–[35]. These results suggest that the axial coordination in oxidized P71H mutant is bis-histidine ligands. On the other hand, as shown in Table S1, the reduced P71H mutant demonstrated almost similar RR absorption to these of the reduced native cyt *c*, indicating that the reduced P71H mutant had Met80/His18 as its axial coordination of heme iron. All results obtained from RR spectra are consistent with the conclusion from UV-visible and CD spectra.

### Sequence-specific assignment

Extensive lists of assignments for yeast cyt *c* in both oxidized and reduced states have been reported in the literatures [35]–[37]. Following the procedures using two dimensional (2D) ^1^H-^1^H total correlation spectroscopy (TOCSY) and correlation spectroscopy (COSY) in H_2_O and D_2_O for spin patterns and 2D ^1^H-^1^H nuclear Overhauser effect spectroscopy (NOESY) spectra in H_2_O for sequential NH-NH and Hα-HN connectivities, we made the assignments for both oxidized and reduced cyt *c* P71H mutant under our experimental conditions (20°C and 50 mM phosphate buffer, pH 7.0). The assignment was begun with the identification of the chemical shifts of residues Gln16, Thr19, Gly29, Asn31 and Leu32 because their spin patterns are resolved outside the diamagnetic envelope. The sequence-specific assignment was successfully performed in the regions of residues −3–17, 20–46, 50–54, 63–69, and 88–103, referencing with the data for the native cyt *c* [35]–[37]. Residues, especially close to the heme and the mutation site, exhibit different chemical shifts, which were finally assigned and given in Table 2. In total, more than 85% of the expected proton resonances had been assigned for the P71H mutant in both oxidized and reduced states, and were deposited in Biological Magnetic Resonance Data Bank (BMRB) under accession numbers 17903 and 17904, respectively.

**Table 2:** New assignments of the oxidized P71H mutant.

residues	chemical shift (ppm)
His^18^	HN 10.59, Hα 8.52, H*β*1 9.17, H*β*2 13.91, H*δ*1 12.19, H*δ*2 19.30, H*ε*1 −11.98
Pro^30^	H*α* 3.93, H*γ* −0.63, H*δ*1 −0.97, H*δ*2 −3.07,
Leu^32^	*δ*1-CH_3_ 0.59, *δ*2-CH_3_ 0.99
Ile^35^	*γ*-CH_3_ 0.17, *δ*-CH_3_ 0.11, *γ*-CH_2_ 0.46
Trp^59^	H*ε* 7.47
Leu^68^	H*α* 3.75, H*β*1 0.47, H*β*2 0.56, H*γ* 0.78, *δ*1-CH_3_ −2.77, *δ*2-CH_3_ −0.99
Met^80^	HN 8.67
Phe^82^	HN 8.70, H*α* 4.42, H*β*1 2.85, H*β*2 3.31
Leu^85^	HN 8.40, H*α* 4.00, H*β*1 0.75, H*β*2 1.10, H*γ* 1.60, *δ*1-CH_3_ −0.05, *δ*2-CH_3_ 0.43
His^71^	HN 11.26, H*α* 10.28, H*β*1 8.78, H*β*2 7.82, H*δ*1 17.00, H*δ*2 30.25, H*ε*1 −14.95
Ala^72^	HN 9.59, H*α* 6.21, *β*-CH_3_ 1.79

### NMR signals assignment of axial ligands and heme of the oxidized P71H variant

**Figure 3:**
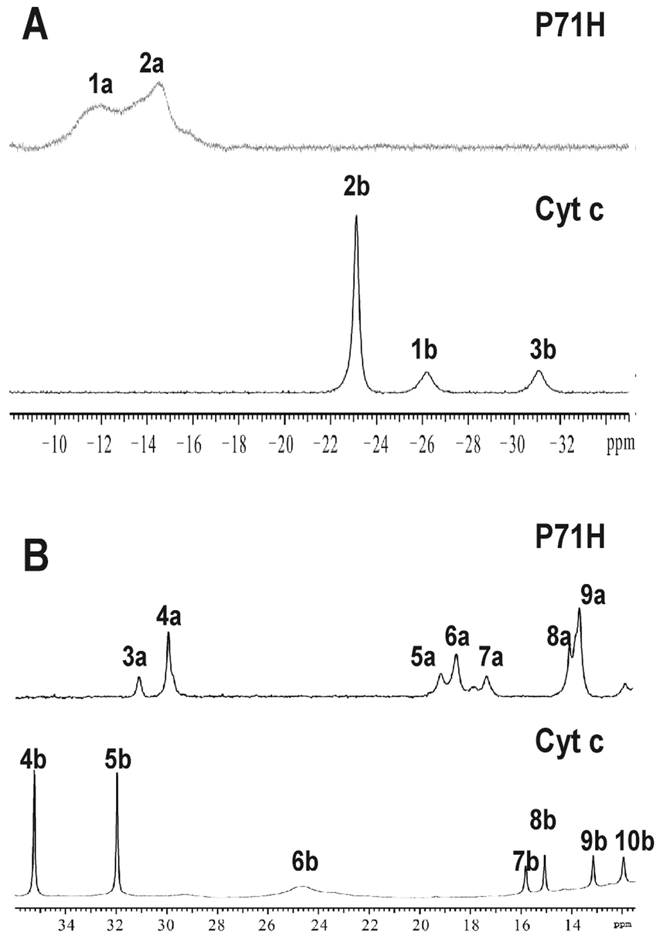
(A) The high-field shifted region of 1D 1H NMR spectra of the native cyt c (down) and its P71H variant (upper) in oxidized state. (B) The down-field shifted region of 1D 1H NMR spectra of the native cyt *c* (down) and its P71H variant (upper) in oxidized state. The peaks in 1D 1H-NMR spectrum were assigned as (1a) His18 Hε1, (2a) His71 Hε1, (3a) His71 Hδ2, (4a) heme 8-CH3, (5a) His18 Hδ2, (6a) heme 3-CH3, (7a) His71 Hδ1, (8a) His18 Hδ1 and (9a) heme 5-CH3, respectively. The peaks in 1D 1H-NMR spectrum of native cyt *c* were assigned as (1b) His18 Hε1, (2b) Met80 ε-CH3, (3b) Met80 Hγ, (4b) heme 8-CH3, (5b) heme 3-CH3, (6b) His18 Hδ2, (7b) heme 7-Hα2, (8b) His18 Hβ2, (9b) heme 7-Hα1 and (10b) His18 Hδ1, respectively.

As shown in Figure 3, the hyperfine shifts of the P71H variant are obviously different from those of native cyt *c*, so heme signals could not be assigned directly based on the similarity of the one dimensional (1D) ^1^H-NMR spectra between the native protein and its mutant. The complete assignments of hyperfine shifted signals of the heme protons, the axial ligands His18 and His71, and the heme bound Cys14 and Cys17 of the oxidized P71H were achieved through analysis of the 2D ^1^H-^1^H NOESY spectra tailored to the relaxation properties of these paramagnetic resonances and through one-dimensional nuclear Overhauser effect (NOE) experiments, as reported previously [35]–[36]. The characteristic peaks at −31.0 ppm (peak 3b) and −22.70 ppm (peak 2b) in the high-field shifted region of 1D ^1^H-NMR spectrum of oxidized native cyt *c* (Figure 3A), which were previously assigned as the axial ligand Met80 H*γ*1 and *ε*-CH_3_ [35]–[36], respectively, were disappeared in the high-field shifted region of 1D ^1^H-NMR spectrum of oxidized P71H mutant. Thus, NMR data also suggested that the Met80 did not serve as axial ligand any more in the oxidized state of P71H mutant.

The signals of the four methyl groups of the heme are well resolved at 29.27, 18.48, 13.62, and 11.27 ppm. The resonances at 29.27 and 11.27 ppm display strong NOE connectivities to a signal at −2.74 ppm. The only proton which is equidistant from two methyl groups is the meso-Hδ, positioned between the 1-CH_3_ and the 8-CH_3_ groups. The signal at 11.27 ppm can be assigned to 1-CH_3_ because it has NOEs with the signals of a thioether group and methyl groups of Leu68 and Leu94, which were also observed in the oxidized native cyt *c*. The assignment of the signal at 29.27 ppm as 8-CH_3_ is consistent with its connectivities with *δ*_2_-CH_3_ of Leu32 (at 0.59 ppm) and ε-CH_3_ of Met64 (at −0.64 ppm). The remaining resonances are assigned to 3-CH_3_ and 5-CH_3_. The resonance at 13.62 ppm is assigned to 5-CH_3_ due to its NOE connectivities to Gly29 Hα (at −0.28 ppm) and Pro30 Hδ (at −0.31 ppm). The resonances of the other heme substituents are assigned by analyzing the NOESY connectivities, starting from position 1 and 3 and “hopping” around the macrocycle (Table 3 and Figure S7).

**Table 3:** The assignment of heme protons of cyt *c* P71H mutant in the oxidized state.

atoms	oxidized native cyt *c* (ppm)	oxidized P71H mutant (ppm)
1-CH_3_	7.57	11.27
2-CH	−0.95	−0.78
2-CH_3_	−2.27	0.14
meso- H*_α_*	2.97	3.59
3-CH_3_	31.92	18.48
4-CH	2.08	1.61
4-CH_3_	2.30	1.53
meso- H*_β_*	−0.42	−0.41
5-CH_3_	10.75	13.62
6- H*_α_*_1_	−1.55	−3.07
6- H*_α_*_2_	2.06	0.62
6- H*_β_*_1_	−0.51	1.51
6- H*_β_*_2_	0.97	2.26
meso- H*_γ_*	7.57	7.54
7- H*_α_*_1_	13.08	10.07
7- H*_α_*_2_	15.74	5.55
7- H*_β_*_1_	−0.20	2.52
7- H*_β_*_2_	1.42	1.31
8-CH_3_	35.19	29.27
meso- H*_δ_*	2.03	−2.74

The peaks at the positions of −26.32 ppm (peak 1b) and 24.54 ppm (peak 6b) (Figure 3) which belong to the side-chain aromatic protons H*ε*1 and H*δ*2 of the axial ligand His18 [35]–[36], respectively, in the oxidized native cyt *c*, are not in the same positions in 1D ^1^H-NMR spectra of the oxidized P71H mutant. The 1D ^1^H-NMR spectra of cyt *c* P71H mutant show broad signals at −11.98 ppm (peak 1a in Figure 3A), −14.95 ppm (peak 2a in Figure 3A), 30.25 ppm (peak 3a in Figure 3B), 19.30 ppm (peak 5a in Figure 3B) and 17.00 ppm (peak 7a in Figure 3B), all of them do not exchange with deuterium in D_2_O solution. The line widths of these signals suggest that they are from protons of the metal ligands, most probably the axial ligands. The saturation of the signal at −11.98 ppm allowed the detection of a NOE with a proton resonating at 12.19 ppm, which displayed NOE connectivities to two H*β* protons of His18 resonating at 13.91 and 9.17 ppm. The assignments of two H*β* protons of His18 are consistent with the NOE patterns between them and the amide HN protons of Leu32 and Thr19 observed in native cyt *c* [35]–[36]. So the signals at 12.19 ppm and −11.98 ppm were assigned as the side chain atoms H*δ*1 and H*ε*1 of His18, respectively. The detectability of the signal H*δ*1 of His18 indicates that its H-bond with Pro30 is preserved in the oxidized cyt *c* P71H mutant. On the other hand, the NOE patterns between the two H*β* protons of His18 and the signal at 19.30 ppm indicate that this signal is from H*δ*2 of His18, which also displays a NOE connectivity with a thioether group at 2-position in heme ring. The saturation of the signal at 10.59 ppm indicates that it has NOE patterns with two H*β* protons of His18, suggesting that it comes from the backbone NH of His18, which is confirmed by the NOE peaks between it and the H*α* of Cys17 and Thr19 HN. The assignment of H*α* of His18 at 8.52 ppm is performed based on NOE patterns between this signal and the HN proton, H*β*2 proton of His18 and between this signal and HN of Thr19.

**Figure 4:**
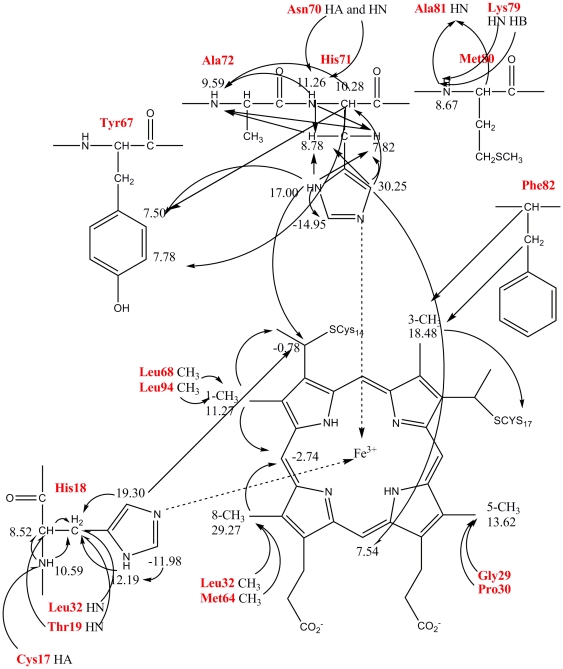
NOE patterns involving the heme and its axial ligands of the oxidized P71H mutant.

With the signals of His18 completely assigned, the remaining broad signals at 30.25, 17.00, and −14.95 ppm should arise from the sixth ligand of the iron. According to our previous results mentioned above, the only reasonable ligand among the unassigned amino acids is His71 in the cyt *c* P71H mutant. Thus, the assignment of the residue His71 was performed based on the relative line widths of the resonances, on the observed NOE patterns and on their comparison to our preliminary structural model. Based on their line widths and chemical shifts, we proposed that the three signals at 30.25, 17.00, and −14.95 ppm were due to the His71 aromatic protons H*δ2*, H*δ*1 and H*ε*1. Saturation of these three signals does not give rise to NOEs with the protons of the known assignments. Moreover, no any scalar connectivities could be detected for any of these signals so that a proton-specific assignment of these signals could be obtained. However, strong NOESY cross-peaks are detectable with almost identical intensity between the signal at 17.00 ppm and unidentified signals resonating at 7.82 and 8.78 ppm, which suggests that the latter two resonances are due to two H*β* protons of His71, the signal resonating at 17.00 ppm is assigned to one of the aromatic protons, nitrogen-attached H*δ*1 or carbon-attached H*δ*2 of His71. The assignment of two H*β* protons of His71 was also confirmed by two cross-peaks between these two resonances and amide proton HN of Ala72 resonating at 9.59 ppm. The broad signal at 17.00 ppm was finally assigned as aromatic proton H*δ*1 of His71, on the basis of weak NOESY cross-peak observed between this signal and that at −14.95 ppm. The broad signal resonating at −14.95 ppm is due to aromatic H*ε*1 of His71 based on three reasons: 1) the proton resonating at negative chemical shift of −14.95 ppm should be very close to heme iron; 2) both the signals with the chemical shifts of −14.95 ppm and 17.00 ppm are out of diamagnetic region, the signal at 17.00 ppm shows NOE patterns with aromatic proton H*δ* (at 7.50 ppm) and H*ε* (at 7.78 ppm) of Tyr67; 3) the signal at 17.00 ppm shows NOE pattern with a thioether group at 2-position in heme ring. The assignment of aromatic H*δ*1 of His71 is confirmed by the weak NOE observed between the signal at 17.00 ppm and that at 10.28 ppm belonging to H*α* proton of His71, the latter assignment of the signal at 10.28 ppm is identified by the NOESY cross-peaks of this signal to two Hβ protons of His71 and HN proton of Ala72. In addition, NOEs are detectable between the signal at 30.25 ppm and the signals resonating at two H*β* protons resonating at 7.82, 8.78 ppm and Hα resonating at 10.28 ppm of His71. This result is an indication that the broad resonance at 30.25 ppm is due to another aromatic proton H*δ*2 of the sixth ligand His71. This assignment is further confirmed by the NOE observed between this signal and meso-H*γ* of heme ring at 7.55 ppm. The assignment of Met80 HN (at 8.67 ppm) is completed according to the NOE observed between this signal and amide protons of Ala81 and Lys79. All connectivities described previously are summarized in Figure 4, where the chemical shifts of some of the hyperfine-shifted resonances are also reported.

### NMR solution structures determination of P71H mutant in both oxidized states

The helical structures of the P71H mutant in both oxidized and reduced states were determined by using reported techniques [35]–[37], characterized by using NOE patterns (Figure S8), are presented in the regions of 3–13, 50–54, 61–69, and 88–103 in the oxidized P71H conformation, while in the segments of 3–13, 51–55, 61–68 and 88–103 in the reduced P71H conformation. They were similar to those presented in the solution structures of the wild-type *Saccharomyces cerevisiae* iso-1 cyt *c* in both oxidized and reduced states [35]–[37], except that helix IV (aa 71–74) in the native cyt *c* were disappeared in both oxidized and reduced states of the P71H mutant.

**Figure 5:**
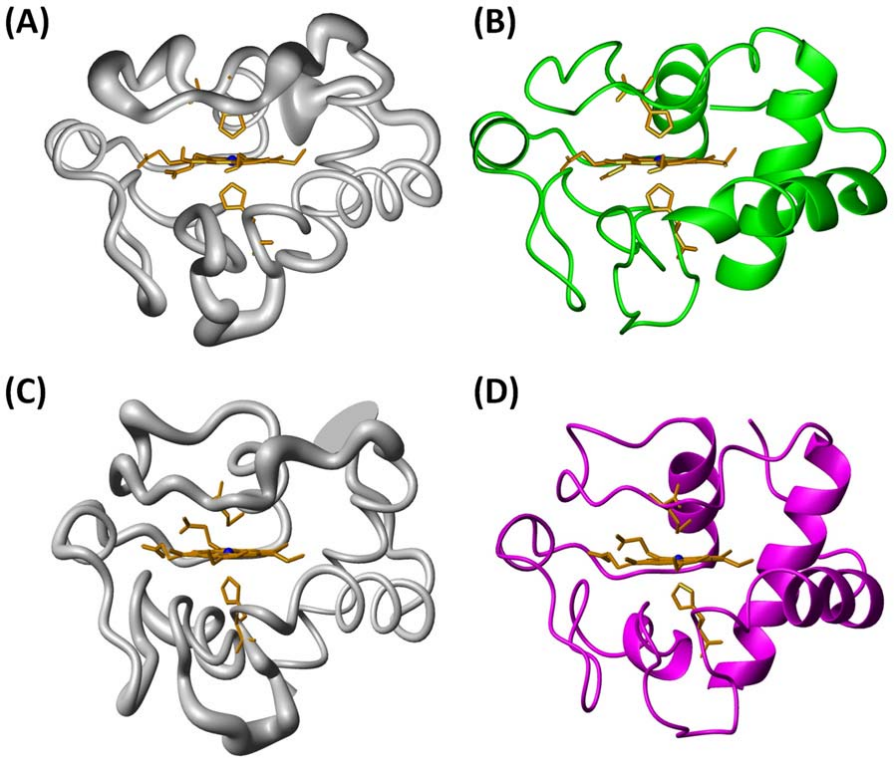
3D solution structures of cyt *c* P71H variant in oxidized and reduced states. (A) Backbone of the family of 20 oxidized P71H structures shown as a tube of variable radius. The radius is proportional to the RMSD of each residue. (B) Ribbon diagram representation of the oxidized P71H mutant. (C) Backbone of the family of 20 reduced P71H structures shown as a tube of variable radius. (D) Ribbon diagram representation of the reduced P71H mutant. The figures are generated with the program MOLMOL. The iron ion was represented as ball.

The solution structures of cyt *c* P71H variant in both oxidized and reduced states were determined by using meaningful and acceptable 1747 and 1788 NOEs, respectively, through the program XPLOR [38]. The heme group, the axial ligands, and the two cysteine (Cys14 and Cys17) covalently attached to the porphyrin are treated as new patch residues. Among them, the patch residue HEC stands for cyt *c*-type heme ring, PHEM is served as one axial ligand histidine of heme iron in the oxidized and reduced P71H mutant, PHMT represents methionine worked as another axial ligand of heme iron in the reduced state of P71H mutant. PHCB and PHCC are used as the residues Cys14 and Cys17. These applications for solution structure calculation of cyt *c*-type heme contained proteins were integrated in the XPLOR-NIH software package. One hundred structures were initially calculated. Upon no NOE violations more than 0.3 Å found, 27 and 25 pairs of hydrogen bond constraints involving slowly exchanging amide protons consistently presented in the initially calculated structures, were introduced as further constraints in the final stage of the structure calculations for oxidized and reduced P71H structures, respectively. Finally, two families, each containing 20 structures with the lowest energy selected from 100 calculated structures, were used to represent the three-dimensional structures of the P71H mutant in oxidized and reduced states, respectively. The conformers of these two bundles showed no NOE violation more than 0.3 Å, and had backbone atoms root mean square deviation (RMSD) values of 0.76±0.14 Å and 0.69±0.10 Å, heavy atoms RMSD values of 1.11±0.14 Å and 1.09±0.14 Å with respect to their corresponding mean structures (calculated for residue −5–103 and heme group), respectively. Figure 5 demonstrated the backbone superimposition of the two families of 20 structures and ribbon representation of three-dimensional solution structures of the P71H mutant in oxidized and reduced states, respectively. The breakdown of experimental constraints per residue was summarized in Figure S9. Both solution structures infer the secondary structures predicted from NOE pattern mentioned early in this paper (Figure S8). The entire structure statistics for these two families of 20 conformers were summarized in Table 4, where 84.5% and 84.6% of the residues were found to be located in the most-favored regions of the Ramachandran plot for the P71H mutant in oxidized and reduced states, respectively. These data indicated that the solution structures of the P71H mutant in both oxidized and reduced states are reasonable.

**Table 4:** Experimental restraints and structural statistics for cyt *c* P71H variant in the oxidized and reduced states.

parameters	20 oxidized structures	20 reduced structures
***Distance restraints from NOEs***		
Total NOE	1747	1788
Intra-residue (i-j = 0)	667	636
Sequential (|i-j| = 1)	320	365
Medium range (1<|i-j|<5)	298	300
Long range (|i-j|>5)	462	487
***H-bond pairs restraints***	54	50
***Structural statistics***		
***r.m.s.d versus the mean structure(Å)***		
All backbone atoms	0.76±0.14	0.69±0.10
All heavy atoms	1.11±0.14	1.09±0.14
Backbone atoms (secondary structure)	0.33±0.09	0.41±0.09
Heavy atoms (secondary structure)	0.78±0.10	0.81±0.09
***r.m.s.d from the experimental restraints***		
NOE distances (Å)	0.031±0.0012	0.024±0.0011
***RMSD from idealized geometry***		
Bonds (Å)	0.0023±0.000012	0.0020±0.000095
Angles (°)	0.34±0.012	0.31±0.011
Impropers (°)	0.51±0.012	0.36±0.017
***Ramachandran analysis*** a		
Residues in most favored regions	84.5%	84.6%
Residues in additionally allowed regions	12.5%	8.8%
Residues in generously allowed regions	3.0%	5.5%
Residues in disallowed regions	0	1.1%
***Number of bad contacts/100 residues*** b	0	0
***Overall G-factor*** b	0.12	0.10

a The programs PROCHECK and PROCHECK-NMR were used to check the overall quality of the structure and GLY and Pro are excluded from the Ramachandran analysis.

b For the PROCHECK statistic, less than 10 bad contacts per 100 residues, and an overall G-factor larger than −0.5 are expected for a good quality structure.

## Discussion

### Analysis of solution structures of the P71H mutant in oxidized and reduced states

**Figure 6:**
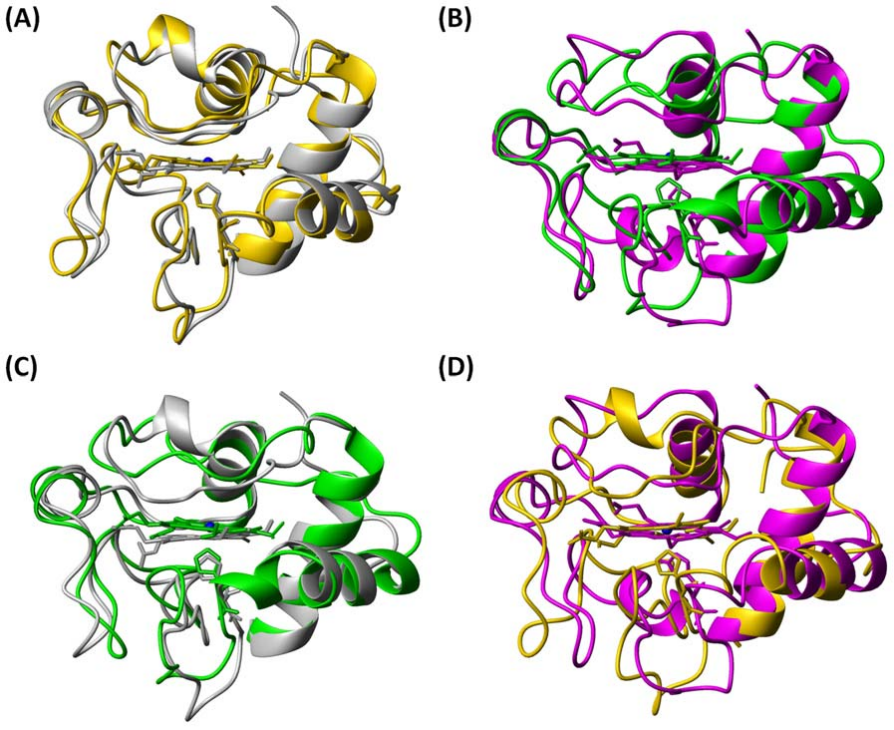
Upon overlaying Cα atoms in second structural region and heme backbone atoms, the conformational comparison: (A) between the oxidized (grey, pdb code 1YIC) and the reduced (gold, pdb code 1YFC) native cyt *c*; (B) between the oxidized (green) and the reduced (magenta) P71H mutant; (C) between the oxidized native cyt *c* (grey, pdb code 1YIC) and P71H (green); (D) between the reduced native cyt *c* (gold, pdb code 1YFC) and the reduced P71H mutant (magenta).

To directly probe the conformer changes in heme moiety relative to heme ring produced by the mutation from Pro71 to His71, in Figure 6, all superimpositions were performed by overlaying backbone C*α* atoms in secondary structural regions and heme ring including all carbon and nitrogen atoms to form the π-conjugate porphyrin system. Obviously, the two conformations of P71H mutant in oxidized and reduced states (Figure 6B) are very different with a global backbone atoms RMSD value of 1.88 Å, while the conformers of native cyt *c* in oxidized and reduced states (pdb codes 1YIC and 1YFC, Figure 6A) were almost same with a global backbone atoms RMSD value of 1.00 Å. Apparently, the coordination between imidazole of His71 side-chain and heme iron in the oxidized P71H mutant made helix III (residues 61–69) shift closer to the heme ring than that in reduced P71H mutant (Figure 6B). Compared to the native oxidized cyt *c*, the conformational switching of the sixth axial ligand from Met80 to His71 in the oxidized protein resulted in loss of helix IV (residues 71–74), as shown in Figure 6C, but the exposure of the heme group to the solvent in the oxidized P71H mutant is almost identical to that of the native oxidized cyt *c* (only 0.9% increment in oxidized P71H variant evaluated with the program MOLMOL, when considering the iron ion and the carbon and nitrogen atoms to form the π-conjugate porphyrin system, similarly hereinafter). In the reduced P71H mutant, the hydrophobic core might be disturbed by the introduction of the hydrophilic residue His71 into the edge of the heme pocket even though its side-chain imidazole does not bind to heme iron. Therefore, the conformers of the reduced native cyt *c* and its P71H mutant have a global backbone RMSD value of 1.90 Å (Figure 6D), much higher than that (1.18 Å) of the conformers of the oxidized native cyt *c* and its P71H mutant (Figure 6C). In the oxidized P71H mutant, the strong coordination between imidazole of His71 and heme iron forces the polypeptide (residues 71–74) closer to heme ring (Figure 6C), so the integrity of the heme hydrophobic pocket is wholly retained. Therefore, in the global folding of cyt *c*, the conserved residue Pro71 was suggested to split the region (aa 60–74) into two helices, and function as a key hydrophobic patch to prevent the heme pocket from the solvent. These significant differences between the solution structures of the native cyt *c* and its P71H mutant were not observed in the X-ray structures of the oxidized states of cyt *c* P71A, P71I, P71S, P71V and P71Nva (Nva is a semi-synthesized amino acid residue) mutants [23], [26], which indicated that there were only small deviations from the native structure even though the bulk of side-chains increased. These mutants were particularly designed to probe functions of Pro71 in folding of the polypeptide of region from residues Asp50 to Cys102. However, because all these structures were not available in PDB, we could not make a further detailed comparison between them and the oxidized P71H mutant.

**Figure 7:**
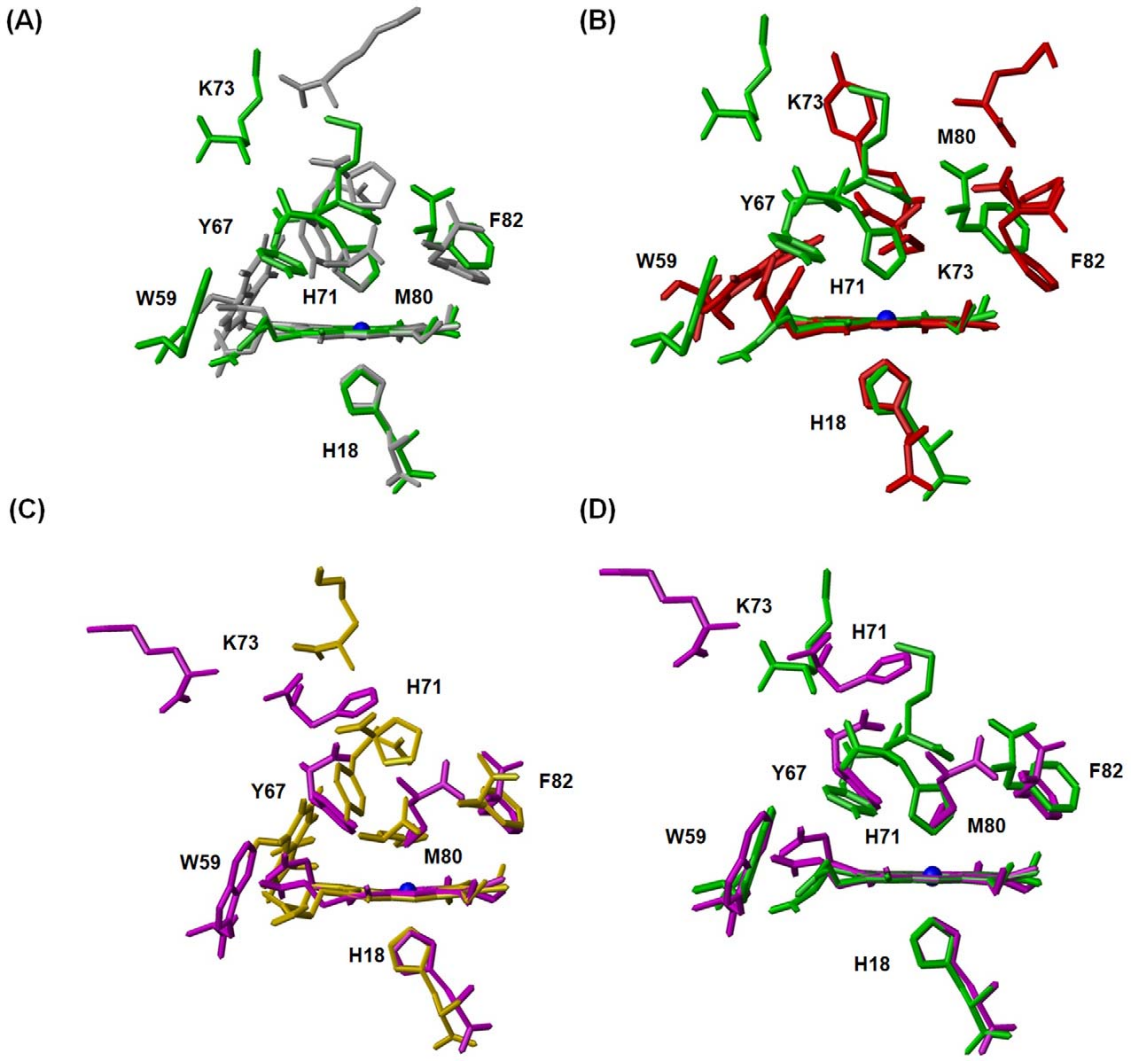
The position comparison of some key residues (including W59, Y67, H71 or P71, K73, F82) relative to heme ring upon overlaying heme ring. (A) between the oxidized native cyt *c* (*grey*, pdb code 1YIC) and its P71H variant (*green*); (B) between the oxidized cyt *c* P71H variant (*green*) and cyt *c* alkaline form (*red*, pdb code 1LMS); (C) between the reduced native cyt *c* (*gold*, pdb code 1YFC) and its P71H mutant (*magenta*); (D) between the oxidized (*green*) and reduced P71H variant (*magenta*).

In addition, the relative positions of some key residues (His18, Trp59, Tyr67, Pro71, Lys73, Met80 and Phe82) to the heme ring were further measured to probe the structural difference among native cyt *c*, its P71H mutant and alkaline comformer by directly superimposing the heme ring (Table S2). As shown in Figure 7, the relative position of side-chain of His18 to heme ring does not change in both oxidized and reduced P71H mutants and even in the cyt *c* alkaline conformer compared to the native cyt *c*. However, in the oxidized P71H mutant, due to coordination between His71 and Fe^3+^ ion, the side-chains of Met80 are forced to shift much farther away from heme ring as well as in the case of cyt c alkaline form (the distances between S atom of Met80 and Fe^3+^ are 11.2 Å and 15.2 Å in oxidized P71H variant and alkaline form, respectively.). Compared to the native oxidized cyt *c*, there are only small changes in the orientation of the side-chain of Tyr67 and the distance between the Tyr67 –OH group and Fe^3+^ ion (4.7 Å in oxidized P71H mutant, while 4.3 Å in native oxidized cyt *c*). However, considering the alkaline form of cyt *c*, as seen in Figure 7B, the distance between the -OH group of Tyr67 and heme Fe^3+^ ion is 15.2 Å. This means that the side-chain of Tyr67 is still pointing to the heme group in oxidized P71H mutant, but in the alkaline form it points outward of the heme pocket. Interestingly, the side-chain of Phe82 keeps its orientation almost unchanged in both oxidized native cyt *c* and its P71H variant, while in alkaline form, it is slightly different from that in oxidized P71H mutant (the distances between Fe^3+^ and C*_β_* atom of Phe82 are 5.7 Å and 7.2 Å in oxidized P71H variant and alkaline form, respectively) (Figures 7A and 7B). The distances between side-chain Nε1 of Trp59 and heme 7-proprionate oxygen are 2.93 Å and 4.95 Å in oxidized native form and its P71H variant, respectively, indicating the hydrogen bond between these two atoms may not exist in the oxidized P71H mutant.

Concerning Lys73 in the native oxidized cyt *c*, the distance between Nζ of Lys73 and Fe^3+^ is 16.9 Å which is a slightly longer than that (16.4 Å) in oxidized P71H mutant resulted from the ligation between His71 and heme Fe^3+^ (Figure 7A). However, the ligation between Lys73 and heme Fe^3+^ in the cyt *c* alkaline form results in large change in the relative position of Lys73, as well as Met80. Obviously, the ligation between Lys73 and Fe^3+^ disturbs the hydrophobic pocket of heme moiety more acutely than that between His71 side-chain and Fe^3+^ ion, indicative of the bigger solvent accessible surface of heme ring in the alkaline form (20% increment compared to the native form)^7^ than that (0.9%) in oxidized P71H variant. Taken together, the cyt *c* P71H mutant can be considered as an intermediate between the native form and alkaline form.

Compared to the reduced native cyt *c* (Figure 7C), the side-chain of His71 in the reduced P71H variant is departed away from the original position of Pro71 side-chain in the reduced native cyt *c* (the distance between N*ε*2 atom of His71 and Fe^2+^ ion is 10.9 Å, the one between C*δ* atom of Pro71 and Fe^2+^ ion is 6.8 Å). Pro71 has a sharp turn towards inside in the reduced native cyt *c*, while His71 imidazole ring in the reduced P71H mutant points outward of heme pocket. The distances between -OH group of Tyr67 and Fe^2+^ ion are 5.0 Å in the reduced native cyt *c* and 4.5 Å in P71H mutant, respectively. The distances between C*β* atom of Phe82 and Fe^2+^ ion are 5.8 Å in both reduced native form and its P71H mutant, and the distances between C*γ* atom of Phe82 and Fe^2+^ ion are 6.1 Å (in reduced native cyt *c*) and 6.5 Å (in the reduced P71H mutant). All these distances measured above suggest that the mutation in Pro71 site result in slight changes in heme moiety in the reduced states, which explains the solvent exposure of the heme group of the reduced P71H mutant remains unchanged compared to that of the reduced native cyt *c* (pdb code 1YFC).

In the oxidized P71H mutant (Figure 7D), the ligation of His71 with Fe^3+^ ion leads to the side-chain of Met80 being released far away from the heme group (the distance between S atom of Met80 and iron ion is 11.2 Å in the oxidized P71H mutant, whereas 2.4 Å in the reduced P71H mutant because Met80 is served as the sixth axial ligand). But the coordination between His71 and Fe^3+^ ion does not change the positions of the side-chains of the residues Phe82 and Tyr67 too much (the distances between -OH group of Tyr67 and Fe^3+^ or Fe^2+^ ion are 4.7 Å and 4.5 Å, the distances between C*β* atom of Phe82 and Fe^3+^ or Fe^2+^ ion are 5.7 Å in oxidized P71H mutant and 5.8 Å in reduced P71H mutant, respectively). Therefore, the conformational switch of the sixth axial ligand from Met80 to His71 only makes helix IV (aa 71–74) disappear, but remains the hydrophobicity of the heme moiety of cyt *c* in both oxidized/oxidation states of P71H mutant.

As we knew, from the native form of oxidized cyt *c* (*i.e.* the conformer III, pdb code 1YCC or 1YIC) to its alkaline form (also called as conformer IV, pdb code 1LMS) [7], [35]–[37], the conformation of the sixth ligand is switched from Met80 in the native form to Lys73 in the alkaline form, a pH dependent conformational toggling, the ligation between side-chain NH_2_ of Lys73 and heme iron also results in the loss of helix IV (aa 71–74). Compared to the alkaline form (Figures S10-A and S10-B), both native cyt c and its oxidized P71H mutant have a global backbone atoms RMSD value 2.2 Å (calculated for residues 9–102aa). The movement of the distal loop in the oxidized P71H mutant is obviously smaller than that in the alkaline form of cyt *c*, but the relative position of helix III (aa 63–69) to the heme ring in the oxidized P71H mutant is a little farther than that in the alkaline form. And the helix II (aa 50–54) in the P71H mutant is almost in the same plane with the heme ring. Obviously, the channel from heme pocket to the solvent in the alkaline form is open larger than that in the oxidized P71H mutant. This result indicated that the ligation between the His71 and heme Fe^3+^ leads to a more packed hydrophobic pocket than that in alkaline form, although the distal loop in oxidized P71H variant looks shifted farther away from heme compared to its native form (Figure 6C).

In summary, the solution structures of cyt *c* P71H mutant demonstrated that the flexible region from Asp50 to Cys102 takes a kind of ‘zigzag riveting ruler structure’, in which the site-directed mutagenesis on Pro71 does not change the secondary structures of cyt *c*, but moves a long distance in the conformation variance of this flexible region. This may be the same mechanism for conformational toggling between the conformer III (the native form of cyt *c* in pH 7.0) and IV (the alkaline form in pH 9.0) [7]. The flexibility in this region facilitated the axial ligand conformation toggling between the conformer III and IV, the surface residue Lys73 turns back to protein interior to coordinate to the heme iron by changing the tertiary structure of cyt *c*, but keeping the secondary structures of cyt *c* unchanged.

### Driving force to switch conformation of the axial ligand

In cytochromes, bis-His and Met/His ligation are more common than the other forms of ligation, where bis-His coordination predominates in the *b*-type cytochromes and Met/His ligation is generally found in the *c*-type cytochromes. However, in this paper, the oxidized yeast iso-1 cyt *c* P71H variant has atypical His18/His71 as its axial ligands, while the reduced P71H mutant presents common Met80/His18 axial ligands. In fact, in nature, these are not the only examples which demonstrated the conformation toggling in different oxidized states. For example, the *E. coli* cytochrome *b*_562_ has atypical Met7/His102 ligation, while the mutation from Met7 to His7 was reported to form a new classic cytochrome *b*-type with bis-His (His7/His102) ligation of heme iron, this mutation has little effect on the *K*_d_ of heme binding but significantly reduces the chemical and thermal stability of the mutant cytochrome relative to its wild-type [39]. CO-sensing transcriptional activator CooA presents Pro2 and Cys75 as its ligands of ferric heme in its oxidized state, while Pro2 and His77 as its ligands of ferrous heme in its reduced state [40]–[47]. The electrochemical redox titration of CooA suggested the formation of the reaction intermediates in the ligand exchange, the ferrous heme with the Pro2/Cys75 coordination, and the ferric heme with Pro2/His77 coordination for the intermediates in reduction and oxidation of CooA [41], [44]. These phenomena suggested that the evolution stress forces the nature to select transition metals to be involved in the proteins. Thus, the metalloproteins were developed by taking the advantages of transition metals capable of possessing different oxidation state and different coordination geometry.

The different preference to ligands of metal ions along with different coordination geometry could be elucidated by the principle of “the hard and soft acid and base (HSAB)” [48]. The harder Lewis acid Fe^3+^ ion prefers to bind the harder Lewis base, the side-chain imidazole ring of His71; while the softer Lewis acid Fe^2+^ ion prefers to associate the softer Lewis base, the S atom of side-chain methyl thioether of methionine; Therefore, the side-chain imidazole of His71 in the oxidized P71H mutant prefers to coordinate with Fe^3+^ ion, while Met80 in the reduced P71H mutant is apt to bind with Fe^2+^ ion with higher affinity than His71. Such extensive conformational changes observed in cyt *c* P71H mutant in different oxidized states for the first time demonstrated how strong these driving forces are and how clever the Nature is, by using the “zigzag riveting structure” (similar to the pantograph device) to fulfill the big moving of residue. His71 (in the cyt *c* P71H variant), His82 (in the cyt *c* F82H variant) and probably Lys73, Lys79 (in the alkaline form of cyt *c*) are those residues in which the affinity of N atoms of these residues to ferric iron of the heme makes ligation between them resulting in big conformational changes.

These features make cyt *c* very stable towards heating, pH and denaturants. On one hand, the structural flexibility of cyt *c* leads reversible ligation of axial ligand among Met80/Lys72/Lys79 based on pH change that protects it properly functioning in the electron transfer process; On the other hand, if the hydrogen network around Tyr67 in heme pocket is disturbed by binding of cyt *c* with membrane (such as cardiolipin, in mitchondria about one third of cyt c are associated with the inner membrane) [49]–[50], cyt *c* by gaining the peroxidase activity goes into the irreversible proapoptotic conformation pathway [51]. Thus, our studies here provided the molecular basis for cyt *c* to be able to carry out a variety of functions based on different conformation.

## Materials and Methods

### Protein overexpression and purification and NMR sample preparation

The mutation from Pro71 to His71 was carried out by using a QuikChange site-directed mutagenesis kit (Stratagene Inc). The native cyt *c* and its P71H mutant were expressed in *E coli* containing the phagemid pBTR2 as described elsewhere [52]. The purification of native cyt *c* and its P71H mutant were performed according to the previously published methods [52]–[54]. The homogeneity of the native and mutant proteins was examined by SDS-PAGE gel electrophoresis and further confirmed by electrospray mass spectrometry. For NMR experiments, about 10 mg of each protein was dissolved in 50 mM aqueous phosphate buffer in 90% H_2_O and 10% D_2_O, and the pH of the solution was carefully adjusted to 7.0. The final concentration was approximately 3 mM in Shigemi NMR tube, determined by using pyridine hemeochrome spectroscopy [55] (Method S2). The sample in D_2_O was prepared by lyophilizing the sample in D_2_O two times and then dissolved in 99.96% D_2_O. The reduced P71H sample for NMR experiments was made by addition of solid *d*_10_-labeled dithiothreitol (DTT) into the solution of oxidized P71H NMR sample up to a final molar ratio of 1∶1, followed by adjusting pH to 7.0.

### UV-visible and Circular dichroism (CD) spectroscopy

The UV-visible spectrum of native oxidized cyt *c* and its P71H mutant were recorded at 25°C on a Hewlett-Packard 8453 diode array spectrophotometer (Figure 2, Figures S2 and S3). The UV-visible absorption of native cyt *c* and its P71H mutant are summarized in Table 1. The far UV CD spectra (Figures S4-A and S4-B) and CD spectra in the Soret region (Figures S4-C and S4-D) were acquired at 25°C on a JASCO J-720 Spectropolarimeter. The CD measurements in the Soret region were obtained using a quartz cell of 1.0 cm path length. The concentrations of the samples used for recording UV-visible and CD spectra were about 2 µM in 50 mM phosphate buffer, pH 7.0. The spectra of reduced proteins were recorded within 2–5 minutes after addition of a small amount of solid sodium dithionite to the ferric protein solution.

### Resonance Raman Spectroscopy

The Ramam measurements were obtained by focusing the output at 413 nm from a Krypton ion laser (Spectra Physics) on the sample jet, as previously described instrumentation [43]. The samples' concentrations were about 2 µM in 50 mM phosphate buffer, pH 7.0.

### NMR spectroscopy

All NMR experiments were acquired at 20°C on a Varian Unity Inova 600 spectrometer with cryo-probe equipped with three channels and pulse-field gradient (where the corresponding ^1^H frequency is 600.2 MHz), or on a Bruker AVANCE III-800 spectrometer with cryo-probe equipped with four channels and pulse-field gradient, where the corresponding ^1^H frequency is 800.2 MHz. To detect connectivities among hyperfine-shifted signals, the 2D ^1^H-^1^H NOESY spectra with a spectral width of 76 ppm in both frequency dimensions [56], with a recycle time of 150 ms and a mixing time of 45 ms, were acquired. To confirm assignment of heme protons, the 1D NOE experiments were performed with the superWEFT pulse sequence and the data connected by using standard methodology [57]. The dipolar connectivities were revealed by two dimensional NOESY experiments. To optimize the detection of connectivities in the diamagnetic region (about −2 ppm to 11.5 ppm), ^1^H-^1^H NOESY spectra were acquired with a recycle time of 1.5 s and mixing times of 50, 100, 150, and 200 ms, respectively. ^1^H-^1^H TOCSY spectra were obtained using the spin-lock times of 30, 50, and 80 ms in H_2_O and D_2_O [58], respectively. ^1^H-^1^H double-quantum filtered COSY (DQF-COSY) spectra were recorded in H_2_O and D_2_O [59]. WATERGATE pulse sequence [60] was used for water signal suppression in NOESY spectra in H_2_O, while in other cases pre-saturation was used. All data consisted of 4K data points in the acquisition dimension and 1K points in the indirect dimension. Raw data were weighted with a squared cosine function, zero-filled, and Fourier-transformed to obtain a final matrix 4096×4096 data points. All spectra were collected at 20°C either on the H_2_O or on the D_2_O samples, processed by using the program NMRPipe [61] and analyzed by the software Sparky 3 (http://www.cgl.ucsf.edu/home/sparky/).

### NMR solution structure calculations

The solution structure calculations were carried out using a standard simulated annealing protocol implemented in the program XPLOR 2.19 (NIH version) [38]. The inter-proton distance restraints derived from NOE intensities were grouped into three distance ranges 1.8–2.9 Å, 1.8–3.5 Å and 1.8–6.0 Å, corresponding to strong, medium and weak NOEs, respectively. Hydrogen bond constraints were introduced based on the un-exchanged backbone amide protons identified in DQF-COSY acquired in D_2_O and the assigned inerratic NOE patterns belonging to the α-helix conformation [25] (Figure S8). The default values for the bond and angle force constants in X-PLOR were employed (500 kcal/molÅ^−2^ and 70 kcal/molÅ^−2^, respectively). A total of 100 structures were calculated, and finally, 20 structures with the lowest energy had no NOE violation >0.3 Å. Structure statistics for these 20 structures were summarized in Table 4. The programs PROCHECK and PROCHECK-NMR were used to evaluate overall quality of the calculated solution structures.

### Accession numbers

The coordinates of the solution structures of cyt *c* P71H mutant in oxidized and reduced states have been deposited with RCSB Protein Data Bank under accession numbers 2lir and 2lit, respectively.

## Supporting Information

Method S1 **Assay of Peroxidase Activity.**
(DOC)

Method S2 **Pyridine hemeochrome spectroscopy.**
(DOC)

Figure S1 **pH dependence of the peroxidase activities of native cyt** ***c*** **and its P71H variant.** The following conditions were used: 100 mM sodium phosphate buffer (pH 6.0–8.0), 100 mM sodium acetate buffer (pH 3.5–6.0); 1 µM protein, 100 µM guaiacol, 200 mM H_2_O_2_.
(DOC)

Figure S2 **UV-visible absorption spectra of wild-type cyt** ***c*** **in the oxidized (solid line) and reduced (dashed line) states at the condition of 100 mM phosphate buffer, pH 7.0.**
(DOC)

Figure S3 (A) UV-visible spectrum of the oxidized cyt *c* P71H variant; (B) The pyridine hemeochrome spectra of the oxidized cyt *c* P71H variant in the oxidized and reduced states in 500–600 nm.
(DOC)

Figure S4 **Far UV CD spectra of oxidized native cyt** ***c*** **(A) and its P71H mutant (B); buffer condition: 20 mM phosphate buffer, pH 7.0 at room temperature.** Soret CD spectra of native cyt *c* and its P71H mutant in the oxidized (C) and reduced (D) states. Conditions: 100 mM phosphate buffer, pH 7.0 at room temperature.
(DOC)

Figure S5 **Resonance Ramam spectra of the oxidized WT cyt** ***c*** **and its P71H mutant.**
(DOC)

Figure S6 **Resonance Ramam spectra of the reduced WT cyt** ***c*** **and its P71H mutant.**
(DOC)

Figure S7 **Atom labeling scheme of the heme moiety in cyt** ***c*** **, and unique NOE contacts between the heme** ***meso*** **-Hs and methyls.**
(DOC)

Figure S8 **Schematic representation of the sequential and medium-range NOE connectivities involving HN, Hα, and Hβ for both (A) oxidized and (B) reduced forms of cyt c P71H mutant.**
(DOC)

Figure S9 **The number of experimental NOEs per residues (A and B) is correlated with global (black) and local (red) backbone RMSD values per residue (C and D) calculated from the 20 structures of the lowest-energy family with respect to the average structure.** Figures A and C are displayed for oxidized P71H mutant, while figures B and D are used to study the reduced P71H mutant.
(DOC)

Figure S10 **The conformational comparison upon overlaying the backbone C** ***α*** **atoms and heme backbone atom:** (A) between the oxidized native form (grey) and the alkaline form (red) of cyt c; (B) between the alkaline form (red) and the oxidized (green) P71H mutant.
(DOC)

Table S1 **Comparison of Raman resonance (RR) modes (cm^−1^) of native cyt c and its P71H mutant. The data of the F82H mutant was cited from supporting reference.**
(DOC)

Table S2 **The measured distances between some key residues and heme ring in different states of cyt** ***c*** **including oxidized and reduced native cyt** ***c*** **and its P71H mutant, and alkaline state of cyt** ***c*** **.**
(DOC)
