# Supplementary material for: Conformational Toggling of Yeast Iso-1-Cytochrome c in the Oxidized and Reduced States
Source: PLoS One. 2011 Nov 8;6(11):e27219. doi: 10.1371/journal.pone.0027219 (PMC3210782; doi:10.1371/journal.pone.0027219)
Supplement: Method S1 — Assay of Peroxidase Activity. (DOC) [file pone.0027219.s001.doc]

**Supplementary data**

**Method S1**

*Assay of Peroxidase Activity* Peroxidase activities of native cyt *c* and its P71H mutant were examined using a characteristic oxidation reaction of *o*-methoxyphenol (guaiacol) to its tetramer [1-2]. The steady-state kinetics were performed with a SF-61 DX2 stopped-flow apparatus (Hi-Tech, UK) thermostatted at 25.0 ± 0.1 ˚C. The H_2_O_2_ solution was freshly prepared with 30% stock solution and its concentration was determined with an absorption coefficient of 39.4 M^-1^cm^-1^ at 240 nm [3]. A solution of cyt c/guaiacol (2 μM and 200 μM, respectively) in a certain pH value buffer and a solution of 400 mM H_2_O_2_ in the same buffer were preincubated at 25.0±0.1 ˚C for 5 min. Then, both solutions were mixed together in the mixing cell of the stopped-flow instrument to start the oxidation reaction. The steady-state reaction rates were obtained by monitoring the absorbance increase at 470 nm. The peroxidase activities of the guaiacol oxidation, between pH 3.5 and 8.0, catalyzed by native cyt *c*, and P71H proteins were shown in Figure S1. The peroxidase activity of the P71H variant is not enhanced by the introduced histidine at position 71, which is lower than that of the native protein. This is due to the fact that His71 occupies the sixth axial ligand site and interferes the access of substrate to the heme iron center.

**Supplementary references**

1. Baldwin D. A., Marques H. M. and Pratt J. M. (1987) Hemes and hemoproteins. 5: Kinetics of the peroxidatic activity of microperoxidase-8: model for the peroxidase enzymes. *J Inorg Biochem*, 30, 203-217.
2. DePillis G. D., Sishta B. P., Mauk A. G. and Ortiz de Montellano P. R. (1991)　Small substrates and cytochrome c are oxidized at different sites of cytochrome c peroxidase. *J Biol Chem*, 266, 19334-19341.
3. Nelson D. P. and Kiesow L. A. (1972) [Enthalpy of decomposition of hydrogen peroxide by catalase at 25 degrees C with molar extinction coefficients of H2O2 solutions in the UV.](http://www.ncbi.nlm.nih.gov/pubmed/5082943) *Anal Biochem*, 49, 474-478.
