# Supplementary material for: Conformational Toggling of Yeast Iso-1-Cytochrome c in the Oxidized and Reduced States
Source: PLoS One. 2011 Nov 8;6(11):e27219. doi: 10.1371/journal.pone.0027219 (PMC3210782; doi:10.1371/journal.pone.0027219)
Supplement: Method S2 — Pyridine hemeochrome spectroscopy. (DOC) [file pone.0027219.s002.doc]

**Supplementary data**

**Method S2**

*Pyridine hemeochrome spectroscopy* Protein concentrations were determined by using the pyridine hemochrome method [1-3], in which, for c-type cytochrome, the protein concentration can be obtained by using the difference in the extinction coefficient constant of reduced cyt *c* protein at 550 nm and 535 nm (ε_R, 550-535_ = 22.1 mM^-1^ cm^-1^). These electronic absorption spectra of the proteins were recorded on a Hewlett-Packard 8453 diode array spectrophotometer. The proteins were dissolved in 50 mM sodium phosphate buffer, pH 7.0, at 25 °C. The temperature was maintained with a circulating water bath equipped with a temperature controller. The spectra of the reduced P71H variant were recorded within one minute after addition of a small amount of solid sodium dithionite (Na_2_S_2_O_4_) to the ferric protein solution. As shown in Figure S2, at the neutral pH condition, the extinction coefficient constant of the P71H variant of cyt *c* was measured as ε_408_ = 133 mM^-1^ cm^-1^, higher than that of wild-type cyt *c* (ε_410_ = 106 mM^-1^ cm^-1^ ), which indicated that the axial coordination of heme iron might be different in wild-type cyt *c* and its P71H mutant.

**Supplementary references**

1. Berry, E. A., and Trumpower, B. L. (1987) Simultaneous determination of hemes a, b, and c from pyridine hemochrome spectra. *Anal Biochem*, 161, 1-15.
2. Wallace, C. J., and Clark-Lewis, I. (1992) Functional role of heme ligation in cyt c. Effects of replacement of methionine 80 with natural and non-natural residues by semisynthesis. *J. Biol. Chem.*, *267*, 3852-61.
3. Lu, Y., Casimiro, D. R., Bren, K. L., Richards, J. H., et al (1993) Structurally engineered cytochromes with unusual ligand-binding properties: expression of Saccharomyces cerevisiae Met-80-->Ala iso-1-cytochrome c. *Proc Natl Acad Sci USA* , 90, 11456-9.
