# Supplementary figures and images for: Conformational Toggling of Yeast Iso-1-Cytochrome c in the Oxidized and Reduced States
Source: PLoS One. 2011 Nov 8;6(11):e27219. doi: 10.1371/journal.pone.0027219 (PMC3210782; doi:10.1371/journal.pone.0027219)

**Supplementary figures**

**Figure S1**:

**
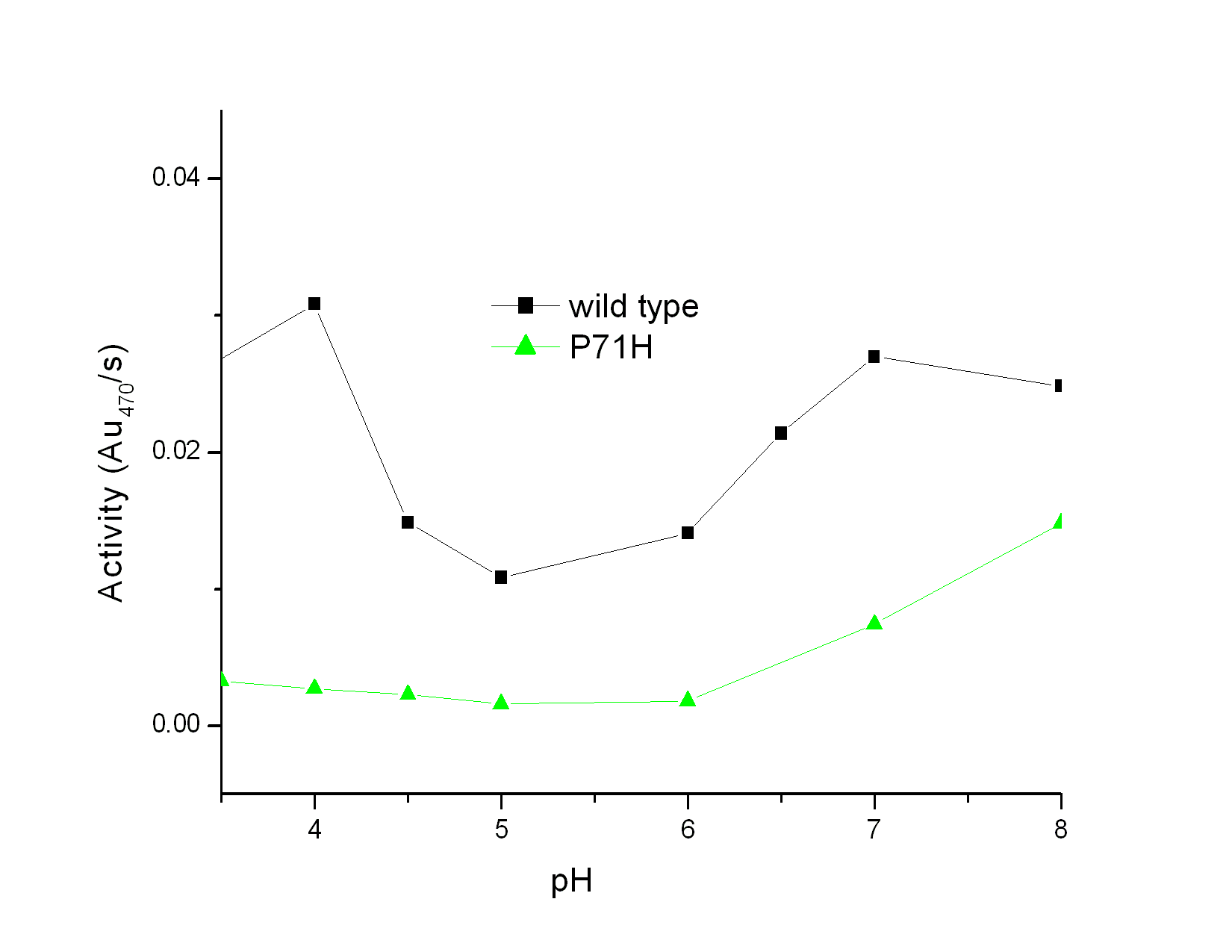
**

Supplement: Figure S1 — pH dependence of the peroxidase activities of native cyt c and its P71H variant. The following conditions were used: 100 mM sodium phosphate buffer (pH 6.0–8.0), 100 mM sodium acetate buffer (pH 3.5–6.0); 1 µM protein, 100 µM guaiacol, 200 mM H2O2. (DOC) [file pone.0027219.s003.doc]

**Supplementary figures**

**Figure S2**

**
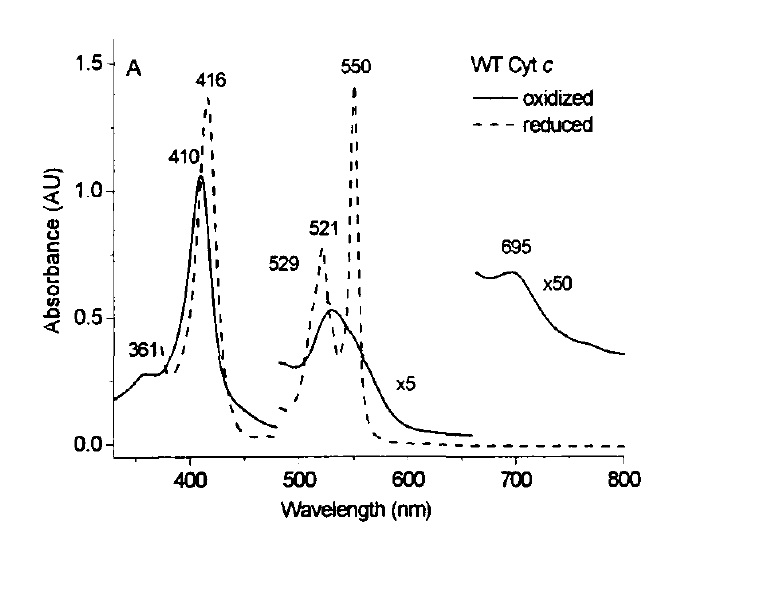
**

Supplement: Figure S2 — UV-visible absorption spectra of wild-type cyt c in the oxidized (solid line) and reduced (dashed line) states at the condition of 100 mM phosphate buffer, pH 7.0. (DOC) [file pone.0027219.s004.doc]

**Supplementary figures**

**Figure S3**


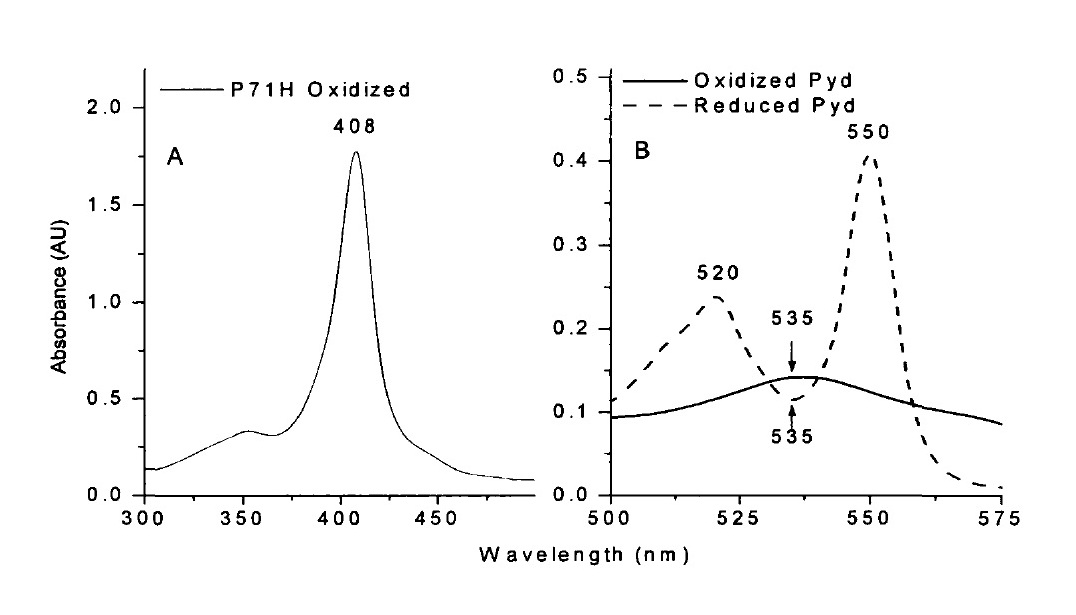

Supplement: Figure S3 — (A) UV-visible spectrum of the oxidized cyt c P71H variant; (B) The pyridine hemeochrome spectra of the oxidized cyt c P71H variant in the oxidized and reduced states in 500–600 nm. (DOC) [file pone.0027219.s005.doc]

**Supplementary figures**

**Figure S4**


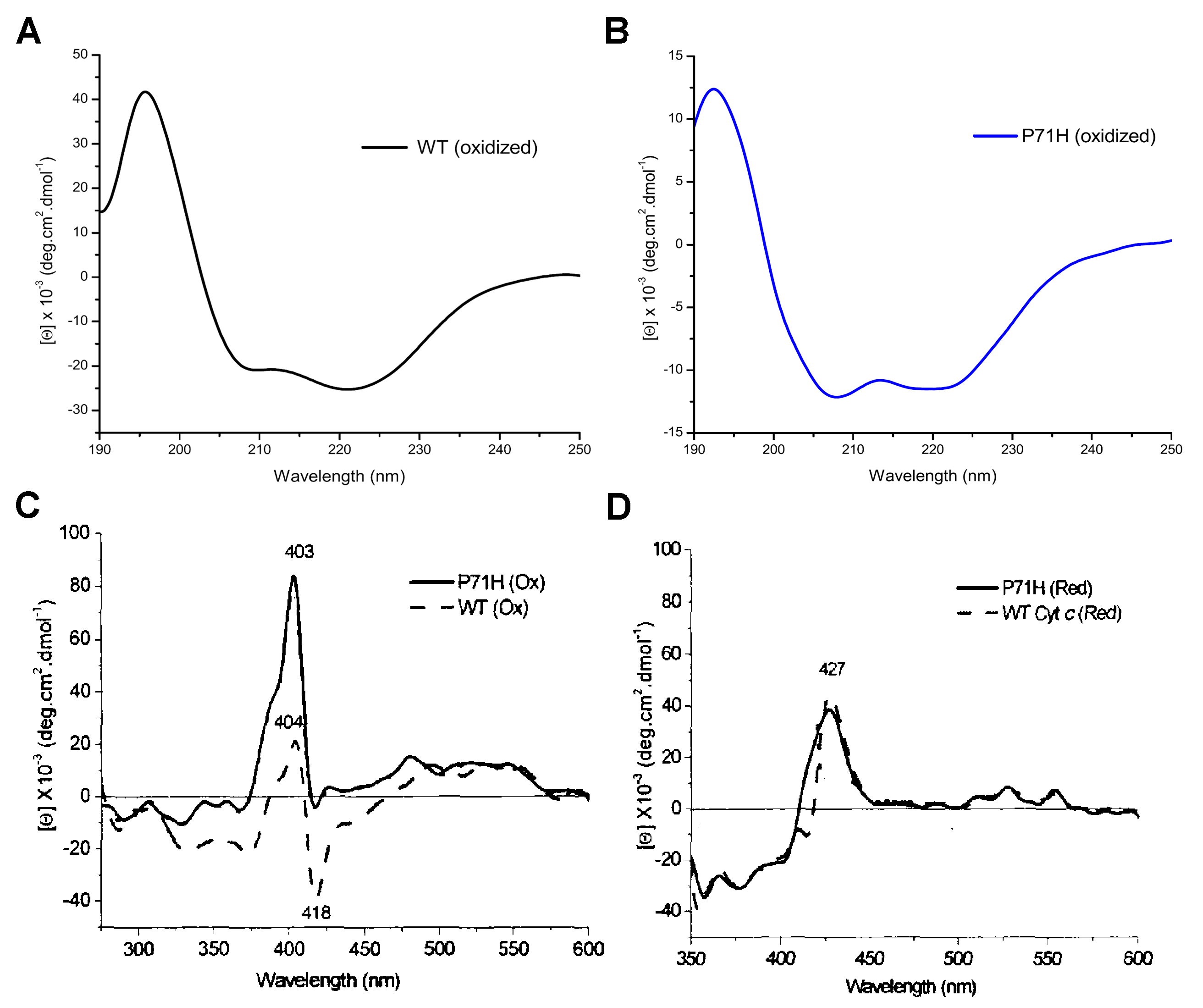

Supplement: Figure S4 — Far UV CD spectra of oxidized native cyt c (A) and its P71H mutant (B); buffer condition: 20 mM phosphate buffer, pH 7.0 at room temperature. Soret CD spectra of native cyt c and its P71H mutant in the oxidized (C) and reduced (D) states. Conditions: 100 mM phosphate buffer, pH 7.0 at room temperature. (DOC) [file pone.0027219.s006.doc]

**Supplementary figures**

**Figure S5**


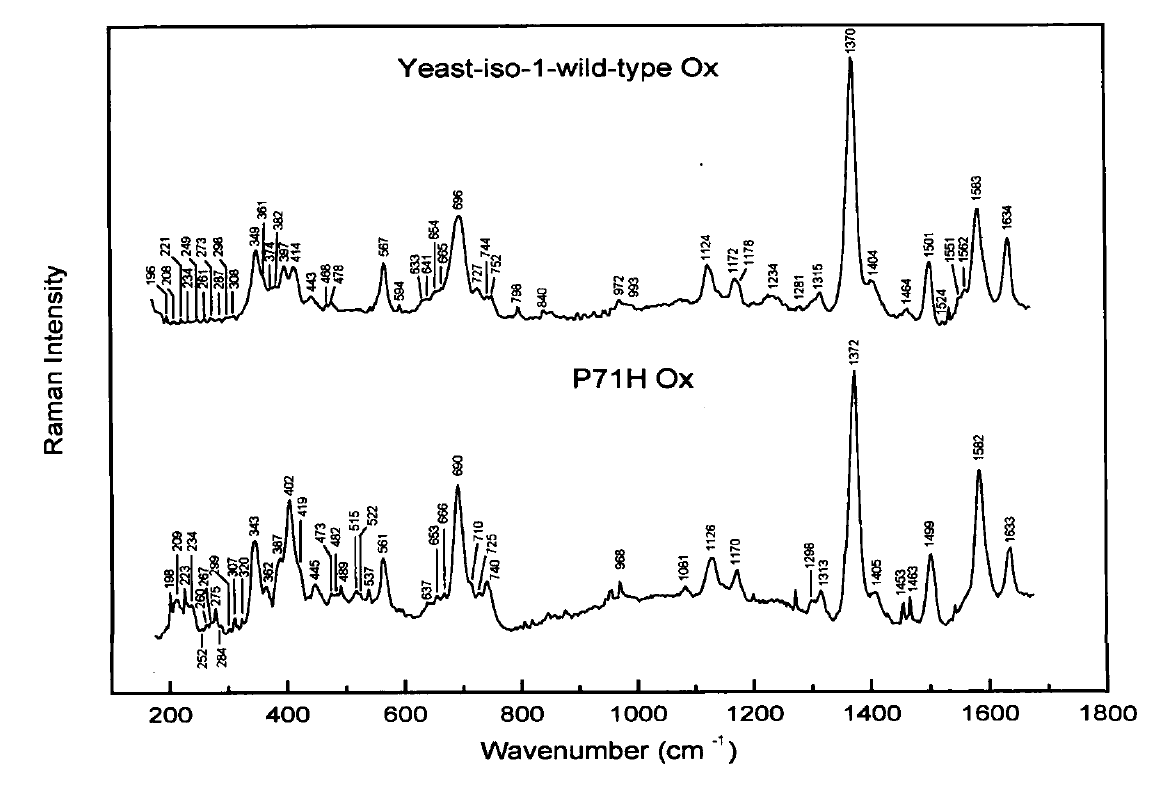

Supplement: Figure S5 — Resonance Ramam spectra of the oxidized WT cyt c and its P71H mutant. (DOC) [file pone.0027219.s007.doc]

**Supplementary figures**

**Figure S6**


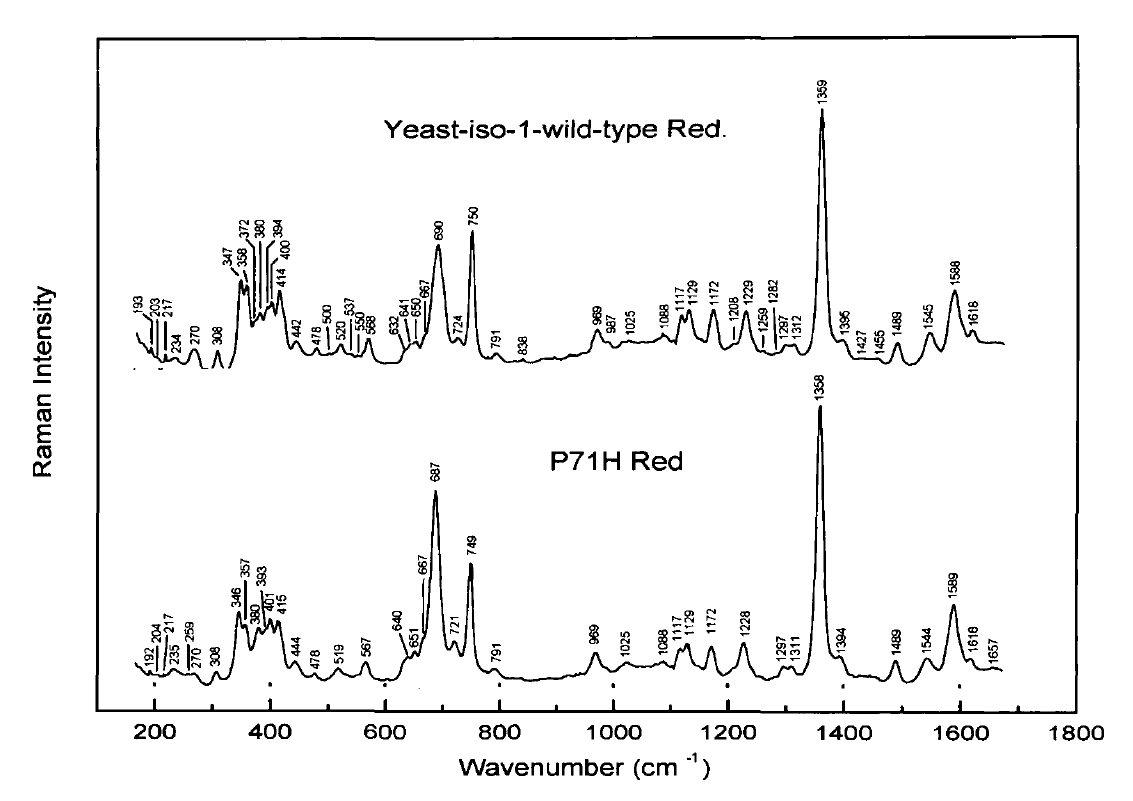

Supplement: Figure S6 — Resonance Ramam spectra of the reduced WT cyt c and its P71H mutant. (DOC) [file pone.0027219.s008.doc]

**Supplementary figures**

**Figure S7**


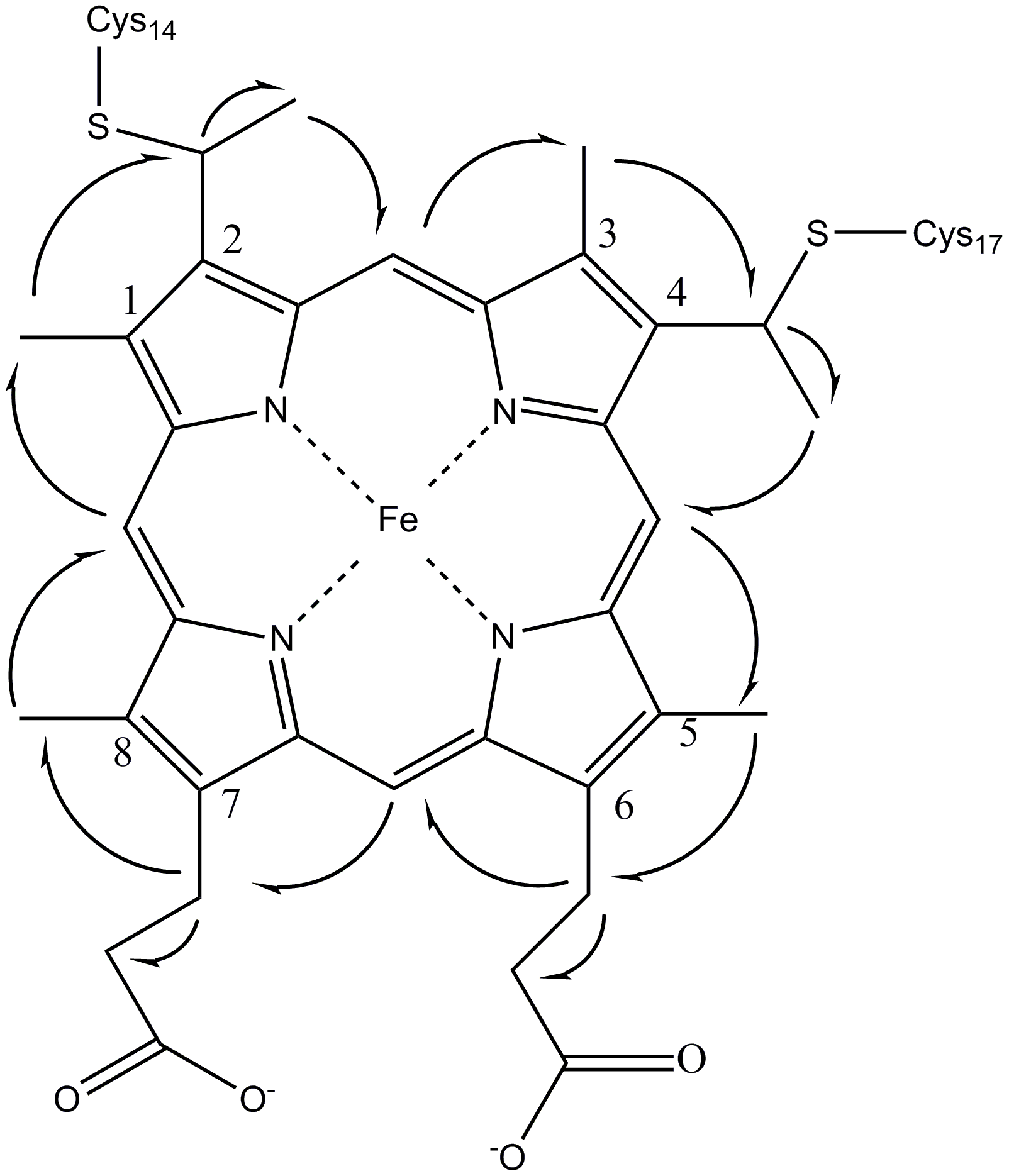

Supplement: Figure S7 — Atom labeling scheme of the heme moiety in cyt c , and unique NOE contacts between the heme meso -Hs and methyls. (DOC) [file pone.0027219.s009.doc]

**Supplementary figures**

Figure S8

**
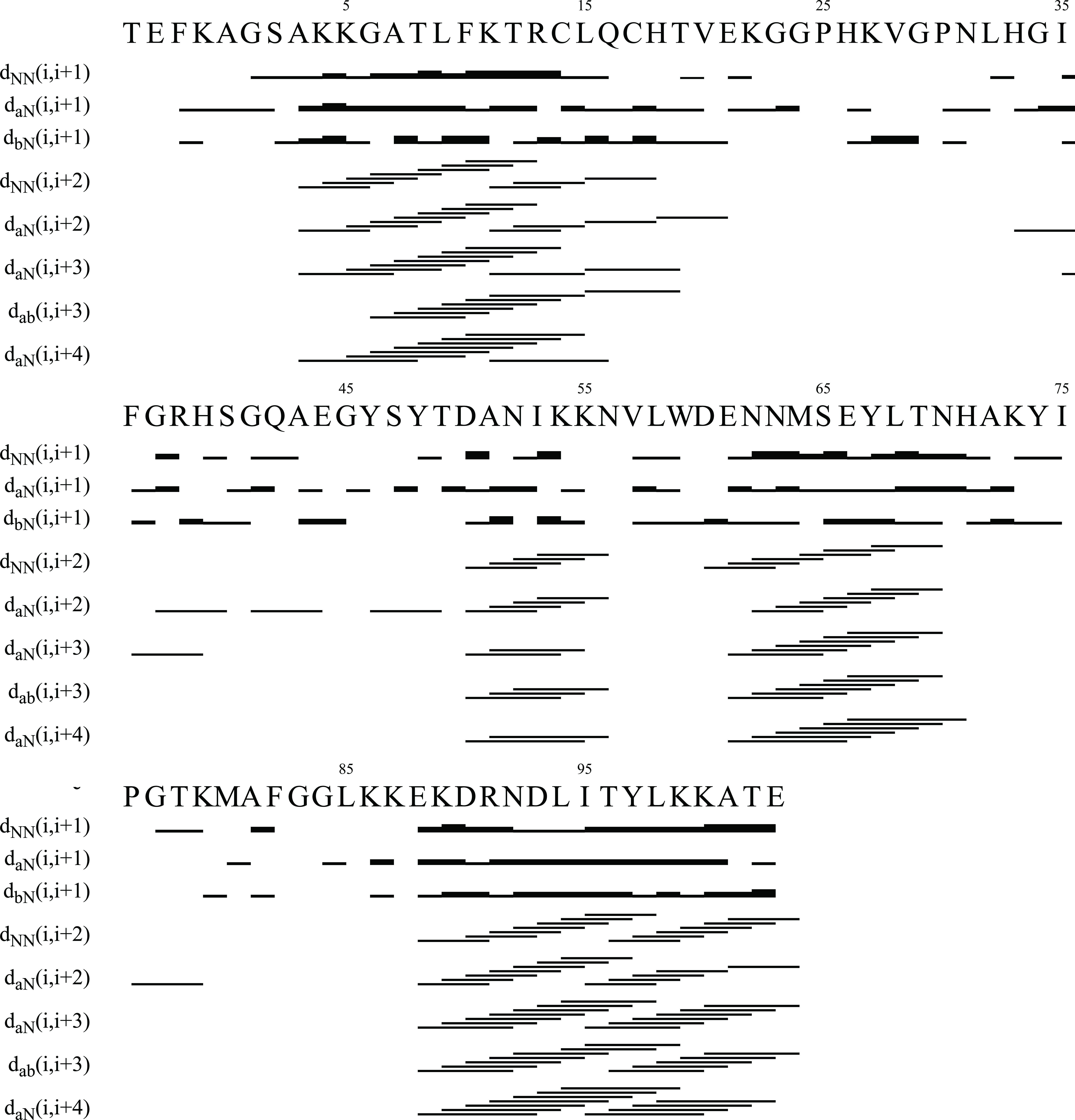
**

Figure S8-A


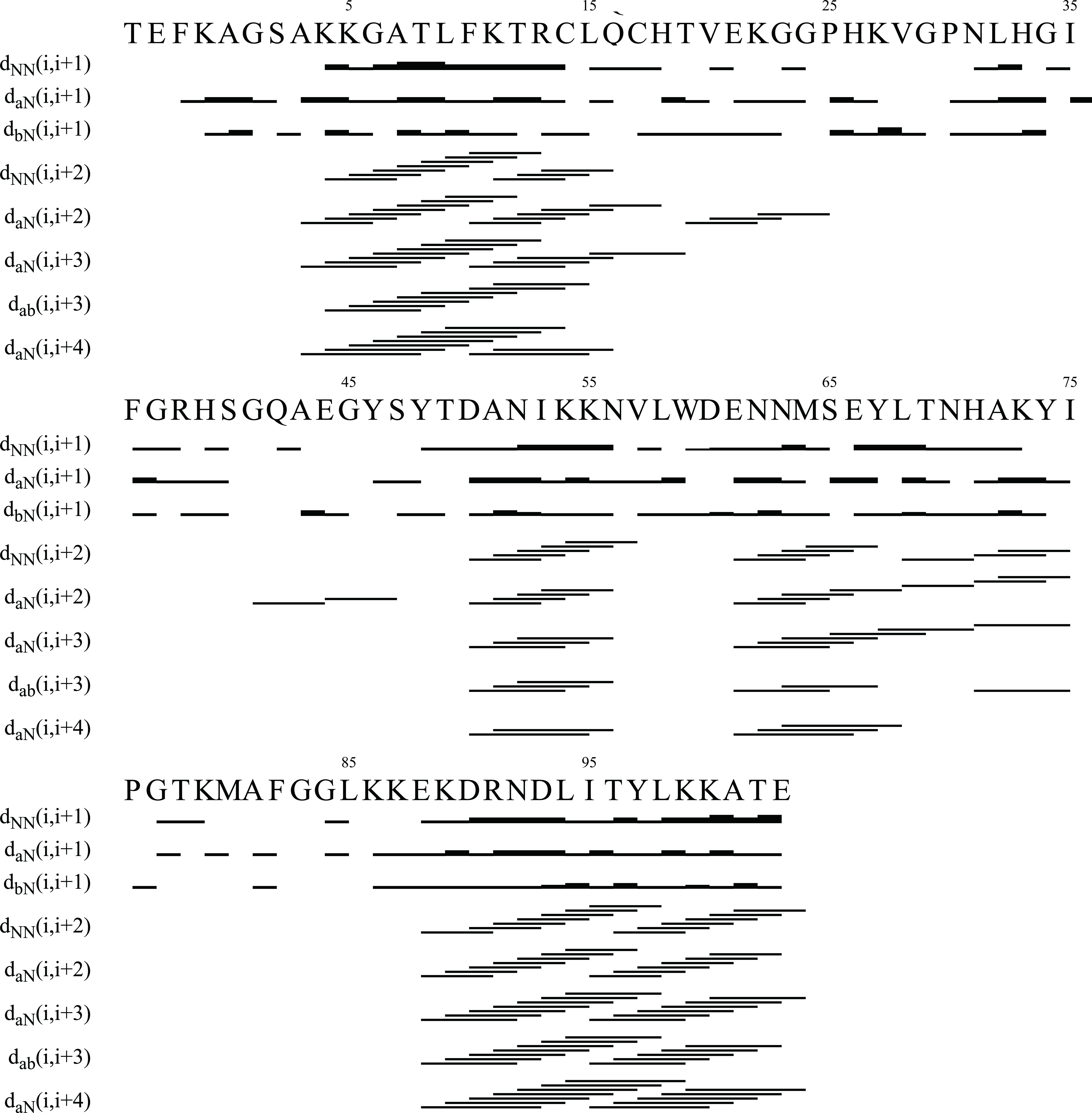


Figure S8-B

Supplement: Figure S8 — Schematic representation of the sequential and medium-range NOE connectivities involving HN, Hα, and Hβ for both (A) oxidized and (B) reduced forms of cyt c P71H mutant. (DOC) [file pone.0027219.s010.doc]

**Supplementary figures**

Figure S9


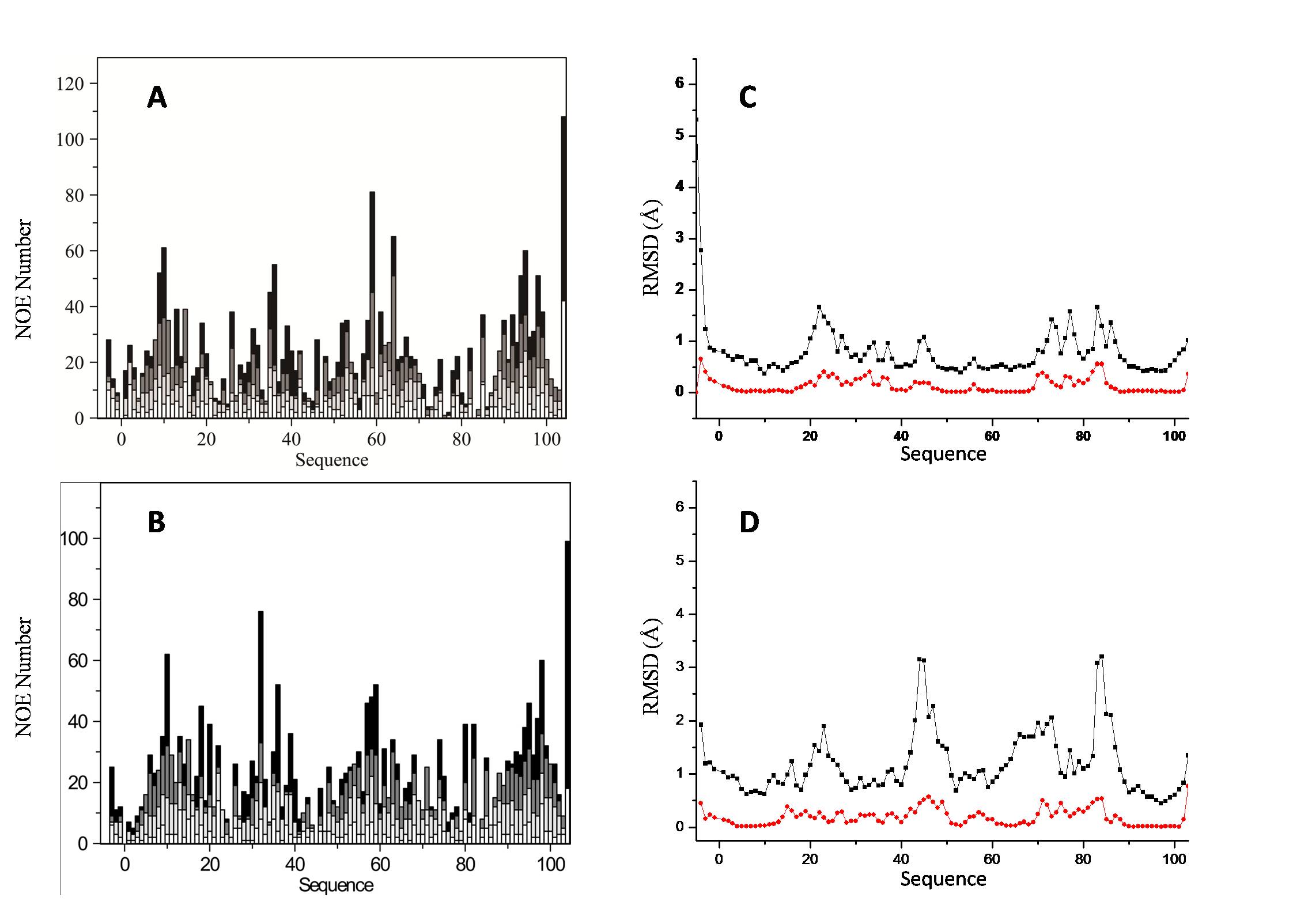

Supplement: Figure S9 — The number of experimental NOEs per residues (A and B) is correlated with global (black) and local (red) backbone RMSD values per residue (C and D) calculated from the 20 structures of the lowest-energy family with respect to the average structure. Figures A and C are displayed for oxidized P71H mutant, while figures B and D are used to study the reduced P71H mutant. (DOC) [file pone.0027219.s011.doc]

**Supplementary figures**

**Figure S10**:

**
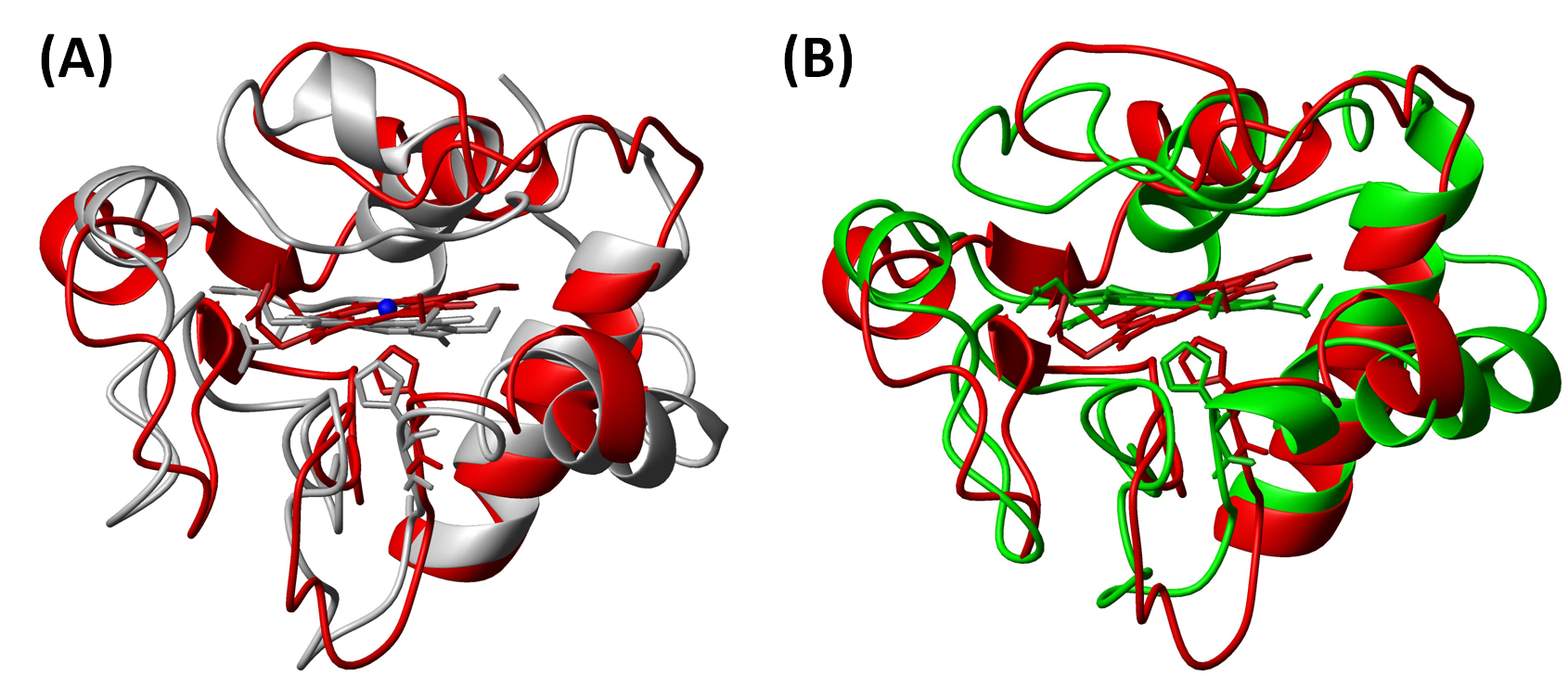
**

Supplement: Figure S10 — The conformational comparison upon overlaying the backbone C α atoms and heme backbone atom: (A) between the oxidized native form (grey) and the alkaline form (red) of cyt c; (B) between the alkaline form (red) and the oxidized (green) P71H mutant. (DOC) [file pone.0027219.s012.doc]
