# Supplementary material for: Conformational Toggling of Yeast Iso-1-Cytochrome c in the Oxidized and Reduced States
Source: PLoS One. 2011 Nov 8;6(11):e27219. doi: 10.1371/journal.pone.0027219 (PMC3210782; doi:10.1371/journal.pone.0027219)
Supplement: Table S1 — Comparison of Raman resonance (RR) modes (cm−1) of native cyt c and its P71H mutant. The data of the F82H mutant was cited from supporting reference. (DOC) [file pone.0027219.s013.doc]

**Supplementary tables**

**Table S1** Comparison of Raman resonance (RR) modes (cm^-1^) of native cyt c and its P71H mutant. The data of the F82H mutant was cited from supporting reference ^1^.

| Modes ^2^ | Oxidized state | | | Reduced state | | |
| --- | --- | --- | --- | --- | --- | --- |
|  | WT | P71H | F82H | WT | P71H | F82H |
| ν_10_ | 1634 | 1633 | 1640 | 1618 | 1618 | 1524 |
| ν_2_ | 1583 | 1582 | 1589 | 1588 | 1589 | 1592 |
| ν_11_ | 1562 | **--** | 1563 | 1545 | 1544 | 1548 |
| ν_3_ | 1501 | 1499 | 1505 | 1489 | 1489 | 1494 |
| ν_29_ | 1404 | 1405 | 1409 | 1395 | 1394 | 1398 |
| ν_4_ | 1370 | 1372 | 1376 | 1359 | 1358 | 1362 |
| ν(C_α_-S) | 696 | 690 | 693 | 690 | 687 | 688 |
| γ_22_ | 443 | 445 | 442 | 442 | 444 | 440 |
| δ(C_β_C_α_S) | 397 | 402 | 404 | 400 | 401 | 401 |
| δ(C_β_C_c_C_d_) | 382 | 387 | 385 | 380 | 380 | 382 |
| ν_50_ | 361 | 362 | 361 | 358 | 357 | 358 |
| ν_8_ | 349 | 343 | 347 | 347 | 346 | 347 |

**Supplementary references**

1. Zheng J., Ye S., Lu T., Cotton TM., Chumanov G., Circular dichroism and resonance Raman comparative studies of wild type cytochrome c and F82H mutant. *Biopolymers* **2000**, 57(2), 77-84.
2. Hu S., Morris IK, Singh JP., Smith KM, Spiro TG. Complete assignment of cytochrome c resonance Raman spectra via enzymatic reconstitution with isotopically labeled hemes. *J Am Chem Soc* **1993**, 115(26), 12446-12458.
