# Supplementary material for: Conformational Toggling of Yeast Iso-1-Cytochrome c in the Oxidized and Reduced States
Source: PLoS One. 2011 Nov 8;6(11):e27219. doi: 10.1371/journal.pone.0027219 (PMC3210782; doi:10.1371/journal.pone.0027219)
Supplement: Table S2 — The measured distances between some key residues and heme ring in different states of cyt c including oxidized and reduced native cyt c and its P71H mutant, and alkaline state of cyt c . (DOC) [file pone.0027219.s014.doc]

**Supplementary tables**

**Table S2** The distances between some key residues and heme ring in different states of cyt *c* including oxidized and reduced native cyt *c* and its P71H mutant, and alkaline state of cyt *c*.

|  | Oxidized  Native cyt c (Å) | Oxidized P71H (Å) | Alkaline  Cyt c (Å) | Reduced native cyt c (Å) | Reduced P71h (Å) |
| --- | --- | --- | --- | --- | --- |
| His18(NE2)-Fe | 1.85 | 2.09 | 1.96 | 1.99 | 2.10 |
| Trp59(Nε1)-HEC(O1A) | 2.93 | 4.95 | 5.76 | 6.30 | 5.78 |
| Trp59(Nε1)-Fe | 9.32 | 10.64 | 9.15 | 9.83 | 11.75 |
| Tyr67(OH)-Fe | 4.30 | 4.71 | 15.22 | 5.01 | 4.52 |
| Tyr67(HH)-Met80(S) | 3.34 | 10.13 | 13.54 | 3.32 | 3.47 |
| Tyr67(OH)-Met80(S) | 3.51 | 9.56 | 12.60 | 3.00 | 3.03 |
| Tyr67(OH)-His71(NE2) |  | 3.86 |  |  | 7.29 |
| His71(NE2)-Fe |  | 2.10 |  |  | 10.90 |
| Pro71(Cδ)-Fe | 9.75 |  | 8.85 | 6.84 |  |
| Lys73(NZ)-Fe | 16.93 | 16.39 | 2.04 | 17.85 | 21.83 |
| Met80(S)-Fe | 2.19 | 11.25 | 15.16 | 2.55 | 2.43 |
| Phe82(Cβ)-Fe | 5.34 | 5.66 | 7.15 | 5.83 | 5.76 |
| Phe82(Cγ)-Fe | 5.68 | 6.52 | 7.04 | 6.08 | 6.52 |
| Phe82(Hζ)-Fe | 8.95 | 8.62 | 9.55 | 8.09 | 8.49 |
